# A cytosolic *mut*p53(E285K) variant confers chemoresistance of malignant melanoma

**DOI:** 10.1038/s41419-023-06360-4

**Published:** 2023-12-14

**Authors:** Luise Dunsche, Nikita Ivanisenko, Shamala Riemann, Sebastian Schindler, Stefan Beissert, Cristian Angeli, Stephanie Kreis, Mahvash Tavassoli, Inna Lavrik, Dagmar Kulms

**Affiliations:** 1 Experimental Dermatology, Department of Dermatology, TU-Dresden, 01307 Dresden, Germany; 2 National Center for Tumor Diseases, TU-Dresden, 01307 Dresden, Germany; 3 Translational Inflammation Research, Medical Faculty, Center of Dynamic Systems, Otto von Guericke University, 39106 Magdeburg, Germany; 4 Department of Life Science and Medicine, University of Luxembourg, Belvaux, 4367 Luxembourg; 5 Molecular Oncology, Guy’s Hospital, Kings College London, London, SE1 1UL UK

**Keywords:** Metastasis, Cell biology

## Abstract

Malignant melanoma (MM) is known to be intrinsically chemoresistant, even though only ~20% of MM carry mutations of the tumor suppressor p53. Despite improvement of systemic therapy the mortality rate of patients suffering from metastatic MM is still ~70%, highlighting the need for alternative treatment options or for the re-establishment of conventional therapeutic approaches, including chemotherapy. Screening the p53 mutation status in a cohort of 19 patient-derived melanoma samples, we identified one rarely described missense mutation of p53 leading to E285K amino acid exchange (*mut*p53(E285K)). Employing structural and computational analysis we revealed a major role of E285 residue in maintaining stable conformation of wild-type p53 (*wt*p53). E285K mutation was predicted to cause interruption of a salt-bridge network affecting the conformation of the C-terminal helix of the DNA-binding domain (DBD) thereby preventing DNA interaction. In this context, a cluster of frequently mutated amino acid residues in cancer was identified to putatively lead to similar structural effects as E285K substitution (E285 cluster). Functional analysis, including knockdown of endogenous p53 and reconstitution with diverse p53 missense mutants confirmed *mut*p53(E285K) to have lost transcriptional activity, to be localized in the cytosol of cancer cells, by both means conferring chemoresistance. Re-sensitization to cisplatin-induced cell death was achieved using clinically approved compounds aiming to restore p53 wild-type function (PRIMA1-Met), or inhibition of AKT-driven MAPK survival pathways (afuresertib), in both cases being partially due to ferroptosis induction. Consequently, active ferroptosis induction using the GPX4 inhibitor RSL3 proved superior in tumorselectively fighting MM cells. Due to high prevalence of the E285-cluster mutations in MM as well as in a variety of other tumor types, we conclude this cluster to serve an important function in tumor development and therapy and suggest new implications for ferroptosis induction in therapeutic applications fighting MM in particular and cancer in general.

## Introduction

MM is considered to be widely chemoresistant due to multiple molecular mechanisms including modulation of the apoptotic machinery [1, 2]. Activating mutations of the serine-threonine kinases NRAS (*mut*NRAS) or BRAF (*mut*BRAF) are key drivers of uncontrolled MM growth through constitutive activation of Mitogen-Activated Protein Kinase (MAPK) pathways RAF-MEK-ERK and PI3K-AKT-mTOR [3–5]. Inhibitors targeting *mut*BRAF and/or downstream MEK have proven high response rates in patients [6, 7], however, the vast majority acquire resistance resulting in tumor relapse. Due to enhanced progression responses of relapsed metastases to immune checkpoint inhibition remain very low [8, 9]. Consequently, MM remains fatal, and demands for alternative treatment options or the reinvention of conventional treatment options, including chemotherapy [10, 11].

Mutation of the tumor suppressor p53 is found in ~50% of all human tumors leading to cancer cell resistance against p53-dependent cell cycle checkpoints and intrinsic apoptosis in response to chemotherapy [12]. In unstressed cells, *wt*p53 is kept at low expression due to MDM2-driven proteasomal turnover. Upon activation *wt*p53 initiates MDM2 transcription in a self-regulatory negative feedback loop [13]. Due to either loss-of-function or gain-of-function missense mutations, p53 remains strongly expressed in tumor cells, because mutants lose recognition of *wt*p53 transcription consensus sequences [14]. In particular missense mutations R175* (* = H), R248* (* = Q, W, G), R249* (* = T), R273* (* = C, L, H, G), R282* (* = W), and G245* (* = S, D) have been identified as hotspots for p53 gain-of-function. They encompass contact mutants that have lost the ability to bind DNA, and conformational mutants which are unable to fold properly showing significantly diminished and/or altered DNA-binding activity [15, 16], in both cases fostering tumor progression and therapy resistance [17]. Hence, restoration of *wt*p53 transcriptional activity [18–20] has become of major interest involving compounds that bind to and convert *mut*p53 into the native form of *wt*p53 (PRIMA1-Met/APR-246) [21], as well as compounds that act as Zn^2+^ chelators intending to inhibit misfolding of *mut*p53 (COTI-2, PEITC; [22, 23]).

Interestingly, only about 20% of MM carry p53 mutations [24], but still present with pronounced chemoresistance. We have recently identified a molecular mechanism by which MM cells that express functional *wt*p53 may escape DNA damage-induced and p53-driven cell death and undergo fast and extensive progression instead [25].

In the present study we screened the p53 mutation status of 19 patient-derived MM samples and identified one p53 missense mutation resulting in E285K conversion [26–28]. Investigating structural as well as functional properties of *mut*p53(E285K) we conclude that it serves a loss-of-function, as it lacks transcription of designated target genes, and confers chemoresistance. We provide evidence that sensitization of patient-derived *mut*p53(E285K)-expressing MM cells to cisplatin via restoration of *wt*p53 function and MAPK inhibition, respectively, involves ferroptosis induction. Hence, we show ferroptosis induction through glutathione peroxidase 4 (GPX4) inhibition to tumorselectively eliminate *mut*p53- as well as *wt*p53-expressing MM cells, paving new avenues for improved MM treatment.

## Materials and methods

### Cells and reagents

Human melanoma cells were isolated from patients metastasis [M#10 = m (63); M#18/M#20 = m (87); M#31 = m (56); M#34 = m (70); M#35 = f (81); M#40 = m (75); M#45 = m (39); M#46/M#51_1/M#51_2/M#53 = m (72/73); M#47_1/M#47_2 = m (72); M#48 = m (52); M#54 = m (74); M#58 = m (82); M#59 = m (48); M#70 = m (51)], through incubation in HBBS (w/o Ca^2+^ and Mg^2+^) containing 0.05% collagenase; 0.1% hyaluronidase; 1.25 U/ml dispase; 20 mM HEPES, 100 g/ml gentamycin; 100 U/ml penicillin and 100 g/ml streptomycin for 60 min at 37 °C in a humified atmosphere of 5% CO_2_, and maintained in RPMI + 10% FCS (Invitrogen, Karlsruhe, Germany). The usage of patient-derived melanoma samples was approved by the ethics committee of the TU-Dresden (SR-EK230052020) and informed consent was obtained from all patients. Human melanoma cell lines WM1552C and SkMel29 were maintained in RPMI 1640 with 10% FCS. Primary human fibroblasts and melanocytes were purchased from Cell Systems (Troisdorf, Germany) and maintained in DMEM + 10% FCS or Melanocyte Growth Medium (M2, Promocell, Heidelberg, Germany).

All cell samples and cell lines were tested every other month to be mycoplasma-negative as judged by the MycoAlert Mycoplasma Detection Kit (LT-07, Lonza, Visp, Switzerland).

For stimulation of cells, Cisplatin (GRY-Pharma, Kirchzarten, Germany) was added at 15 µM, dabrafenib (#S2807; Selleckchem, Munich, Germany) at 10 µM, trametinib (#S2673; Selleckchem) at 10 nM, QVD (#IMI-2309-1; Novus Biologicals, Littleton, CO, USA) at 5 µM, Nec1s (#2236; BioVision, Hannover, Germany) at 15 µM, alpelisip (#HY-15244; MedChemExpress, Monmouth Junction, NJ, USA) at 10 µM, afuresertib (#S7521; Selleckchem) at 10 µM, PRIMA1-Met (#HY-19980; MedChemExpress) at 5–40 µM, COTI-2 (#HY-19896; MedChemExpress) at 0.5–10 µM, PEITC (#253731; Sigma-Aldrich, Taufkirchen, Germany) at 1–15 µM, MG132 (Merck Millipore, Darmstadt, Germany) at 10 µM, ferrostatin-1 at 15 µM, and RSL3 at 1.13 µM (kindly provided by Andreas Linkermann, TU-Dresden) to cell culture media.

### RT-PCR, plasmids, cloning and transfection

RNA was extracted from 2 × 10^6^ cells, reverse transcribed using First Strand cDNA Synthesis Kit (Thermo Scientific, Waltham, MA, USA) and subjected to PCR amplification using primers directed against p21 and MDM2 (#HP200369; #HP206085, OriGene Technologies, Inc. Rockville, MD, USA). GAPDH (forward: 5′-GCCTCCTGCACCACCAACTGC-3′ and reverse primer: 5′-CCCTCCGACGCCTGCTTCAC-3′) served as housekeeping expression control. For cloning and sequencing p53 was reverse transcribed and amplified via PCR using forward: 5′-CTAGCTAGCATGGAGGAGCCGCAG-3′ and reverse primer: 5′-GCATCTAGAGTCTGACTGAGGCCCTTC-3′. cDNA was cloned via NheI/XbaI restriction into pcDNA3.1(+) and subjected to sequence analysis (GATC, Konstanz, Germany) using pcDNA3.1-FP 5′-CTCTGGCTAACTAGAGAAC-3′ and pcDNA3.1-RP 5′-CAAACAACAGATGGCTGGC-3′ primer within the vector, and p53for 5′-ATGACGGAGGTTGTGAG-3′ and p53rev 5′-ACTCGGATAAGATGCTGAGG-3′ primers designed to match with the p53 cDNA. Sequences were subjected to BLAST analysis compared to *wt*p53 transcript variant 1 (https://www.ncbi.nlm.nih.gov/nuccore/NM_000546.4). pRetroSuper-blasto-p53i plasmid was used to stably knock down p53 in the presence of 5 µg/ml blasticidin (Thermo Scientific) [29]. Ectopic re-expression of p53 was facilitated by electroporating 6.5 × 10^6^ cells with 25 µg of pCMV-neo-Bam-based p53 variants encoding plasmids containing silent mismatches [29] in 600 µl RPMI + 10% FCS + 1.25% DMSO. Based on the pCMV-neo-Bam-*wt*p53 plasmid site directed mutagenesis was performed to gain p53-E285K, p53-E285R, p53-K132E, p53-R175H, and p53-R248W mutants. Pfu-ultra polymerase (Promega, Madison, VC, USA) followed by *DpnI* digestion (Thermo Scientific) was used according to the manufacturer’s instruction.

### 3D melanoma spheroids

M#31 and M#54 cells were transfected with pEGFP-N1 and stable clones selected by sorting (FACSAria III, BD-Biosciences, Heidelberg, Germany). Spheroids were generated using the “hanging drop” method as described before using 20 × 10^4^ cells per drop [30]. After 12 days, individual spheroids were embedded into 30 µl of a dextran-based gel-matrices containing 4 nMol/L of thiol-reactive groups (3-D Life Dextran-CD Hydrogel SG, #G93-1; Cellendes, Reutlingen, Germany), according to the manufacturer’s protocol [30] and incubated at 37 °C for 30 min. Subsequently, gels were covered with medium. Cell death of spheroids was visualized by addition of 6.7 µg/ml propidium iodide (PI, #3566, Thermo Scientific) in PBS for 20 min at RT. Confocal images were taken using an LSM 780/FCS inverse confocal fluorescence microscope (Zeiss, Marburg, Germany). Green fluorescence emission peak was 488 nm (emission filter 499–597 nm), 561 nm for red fluorescence (emission filter 606–686 nm).

### Determination of cell death

Cell death was quantified in a 96-well format by determining PI (1 µg/ml) uptake every 4 h for 24 h using automated image-based IncuCyte® (Satorius, Goettingen, Germany) screening technology. High red intensity was quantified using the “cell-by-cell-module”.

### TUBE assay

Cells were lysed in TUBE lysis buffer (50 mM Tris-HCl, pH 7.5; 150 mM NaCl; 1% NP-40, 1 mM EDTA 10% glycerol) supplemented with Complete® (Roche, Mannheim, Germany), 10 µM PR-619 and 1 × 1,10-phenoanthroline. TUBE-Agarose pull down was performed according to the manufacturer’s instructions (UM401, Life Sensors, Philadelphia, PN, USA), and protein extracts analyzed by Western-blotting.

### Western-blot analysis

For whole cell lysates, cells were lysed in lysis buffer (50 mM HEPES, pH 7.5; 150 mM NaCl; 10% glycerol; 1% Triton-X-100; 1.5 mM MgCl_2_; 1 mM EGTA; 100 mM NaF; 10 mM pyrophosphate; 0.01% NaN_3_ (phosSTOP®; Complete®). For fractionation cytosolic (C: 10 mM HEPES, pH 7.5; 10 mM KCl; 100 µM EDTA; 0.5% NP-40) and nuclear (N: 20 mM HEPES, pH 7.5; 400 mM NaCl, 1 mM EDTA) extraction buffers were supplemented with phosSTOP®; Complete®; 1 mM DTT and 1 mM PMSF. Protein content was determined using DC Protein assay kit (BioRad, Hercules, USA). Protein extracts were subjected to SDS-PAGE (BioRad), blotted onto nitrocellulose membranes and incubated with antibodies directed against PARP, p53 (#551025, #554293, BD-Biosciences), caspase-3, cleaved caspase-3, γH2AX, H3, IκBα, MDM2, p21, p-p53(Ser15), PUMA, Tom20, and ubiquitin (#9665, #9661, #2577, #4499 #4814, #86934, #2947, #9284, #12450, #42406, #43124, Cell Signaling, Camebridge, UK), respectively. Equal loading was monitored by 0.1% Ponceau S (#5983.2, Roth, Karlsruhe, Germany) staining, and/or by re-probing membranes with an antibody against β-actin (#4970, Cell Signaling). HRP-conjugated secondary antibodies (mouse: #NA931; rabbit: #NA934) were purchased from GE-Healthcare (Buckinghamshire, UK). Bands were visualized with chemiluminescense SuperSignal^®^ detection systems (Thermo Scientific).

### Quantification and statistical analysis

Unless stated otherwise, results of cell death analysis are presented as mean ± SD of 3 independently performed experiments. PI intensity of respective control cells was subtracted from PI intensity in response cisplatin treatment to gain net increase in PI uptake, as shown in graphs. Western-blot analysis, RT-PCR, and immunofluorescent images represent one out of 3 independently performed experiments. Statistical analysis was performed with unpaired Student *t-test* using GraphPad PRISM 6 software (https://www.graphpad.com). Quantification of tumor mass was performed calculating the area of the green fluorescent spheroid and the red PI stained cells using Fiji software (https://fiji.sc).

### Bioinformatics and structural modeling

The ranking of solid cancers was deduced from https://gco.iarc.fr/today, excluding non-solid tumors (Non-Hodgkin lymphoma and leukemia) (Table S1). 10 pan-cancer studies from https://www.cbioportal.org were used to determine the frequency of p53 mutations in the selected cancer types. For analysis of p53-specific mutations in melanoma, data from https://www.cbioportal.org/study/summary?id=msk_met_2021 [31] were evaluated [32, 33]. The TP53 Database https://tp53.isb-cgc.org [34] was used to analyze the prevalence of p53 mutation in individual cancer types.

The structure of full-length *wt*p53 was derived from the AlphaFold Database [35]. The structure of *mut*p53 DNA-binding domain was predicted with ESMFold model [36]. The structural model of *mut*p53 bound to DNA was derived by structural alignment and replacing the *wt*p53 subunit from *wt*p53/DNA complex [37] by ESMFold-derived models using PyMOL software (https://pymol.org/2/). Stability changes were estimated as the per-residue difference in the pLDDT score derived from ESMFold models between wild-type and mutant proteins. Rosetta energy of interactions was predicted using RosettaDDG implemented within the PyRosetta framework [38] and ESMFold-derived model of p53 as an input. Stability changes using PoPMuSiC [39], PremPS [40], and MAESTRO [41] were predicted through corresponding web interfaces. The crystal structure of the p53 DNA-binding domain (PDB ID 2ADY) [42] was used for predictions.

## Results

### Malignant melanoma comprises about 20% p53 mutations

**Fig. 1:**
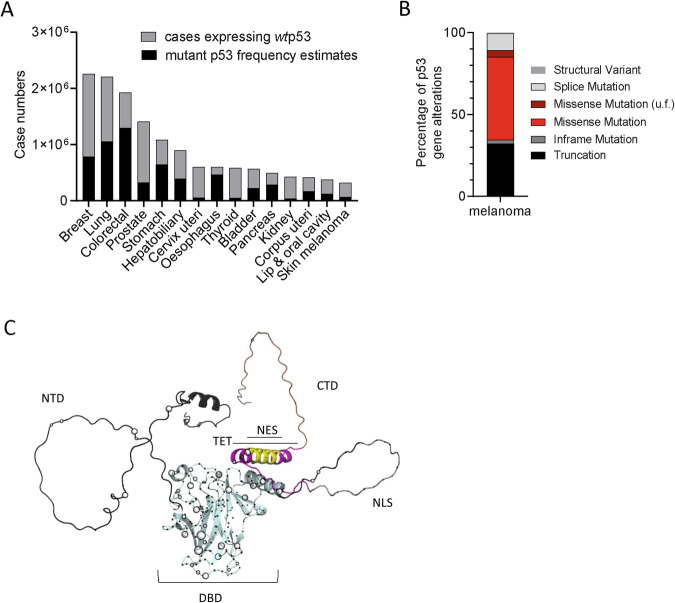
Malignant melanoma comprises about 20% p53 mutations. **A** The frequency of p53 mutations taken from https://www.cbioportal.org was multiplied by the case number of the selected cancers https://gco.iarc.fr/today to get the estimated cases of mutant p53. **B** Percentage of different p53 alterations in melanoma analyzed from https://www.cbioportal.org/study/summary?id=msk_met_2021. **C** Full atom p53 structure with positions of mutations. NTD (1-93) = Intrinsically Disordered N-terminal Domain (gray); CTD (360-393) = C-terminal domain (brown); NES (340–351) = nuclear localization signal (yellow); TET (323–360) = tetramerization domain (purple); DBD (94–294) = DNA-binding domain (light blue). Spheres indicate the positions of missense mutations observed in tumor samples according to https://tp53.isb-cgc.org. The radii of spheres correspond to the mutation frequency in cancer.

MM was recently ranked among the top 15 most prevalent solid tumors worldwide (https://gco.iarc.fr/today; Fig. 1A; Table S1). While mutation of the tumor suppressor p53 presents with highest incidence among all cancers (48.3%), MM comprises only 21.5% p53 mutations (https://www.cbioportal.org; [32, 33] Fig. 1A; Table S2A, B), despite showing a highly progressive phenotype and chemoresistance. The spectrum of p53 mutations shows high diversity, predominantly truncations and missense mutations as deduced from https://www.cbioportal.org/study/summary?id=msk_met_2021 [31] (Fig. 1B). Most missense mutations as identified by *The TP53 Database* (https://tp53.isb-cgc.org; [34]) are located within the DBD of this transcription factor as indicated by spheres within the AlphaFold database-derived p53 protein structure model [35] (Fig. 1C).

### p53 E285K mutation is localized close to the DNA-binding domain and renders melanoma cells resistant to cisplatin-induced cell death

To investigate the impact of *wt*p53 *versus mut*p53 on therapy responsiveness we sequenced p53 of 19 melanoma cell samples isolated from 14 patients with known BRAF/NRAS mutation status, and therapeutic record. In particular, from three primary tumors (Table 1, group 1 = light gray), seven systemic metastases (Table 1, group 2 = gray), and nine brain metastases (Table 1, group 3 = dark gray). In each group at least one p53 variant was identified: primary tumor-derived M#34 presented a c.237-238 deletion resulting in frame shift and premature stop at codon 147; skin metastasis-derived M#35 was identified to be a splice variant; brain metastasis-derived M#10 showed genomic loss of p53, all of which represent p53 loss-of-function. Brain metastasis-derived M#31 represented the only c.853G > A missense mutation, resulting in E285K amino acid exchange (Table 1).

**Table 1:** Overview of patient-derived tumor sample analysis.

M#	Mutation status	Localization	Therapy	p53 status
M#18	NRASQ61H	Primary tumor	None/surgery	wt (P72R)
M#34	NRASQ61L	Primary tumor	None	Truncated (cod. 147)
M#59	BRAFV600E	Primary tumor	None	wt
M#20	NRASQ61H	Lymph node metastasis	None/surgery	wt (P72R)
M#35	NRASQ61L	Skin metastasis	None	Splice mutation
M#45	BRAFV600E	Muscle metastasis	IFNα/ipilimumab/surgery	wt (P72R)
M#47_1	NRASQ61L	Skin metastasis	None	wt (P72R)
M#47_4	NRASQ61L	Skin metastasis	None	wt (P72R)
M#51_1	BRAFV600E	Lymph node metastasis	Dabrafenib/trametinib	wt
M#51_2	BRAFV600E	Intestine metastasis	Dabrafenib/trametinib	wt
M#10	BRAFV600E	Brain metastasis	IFNα/dabrafenib	Genomic loss
M#31	NRASQ61L	Brain metastasis	IFNα/pembrolizumab/bevazicumab/IFNα	E285K
M#40	wt	Brain metastasis	Nivolumab	wt (P72R)
M#46	BRAFV600E	Brain metastasis	None	wt
M#48	NRASQ61R	Brain metastasis	Ipilimumab/nivolumab	wt (P72R)
M#53	BRAFV600E	Brain metastasis	Dabrafenib/trametinib	wt
M#54	wt	Brain metastasis	Ipilimumab/nivolumab	wt (P72R)
M#58	wt	Brain metastasis	None	wt
M#70	BRAFV600K-NRASQ61R	Brain metastasis	Vemurafenib/cobimetinib	wt (P72R)

P53 mutations status of 19 human melanoma cell samples isolated from primary tumor (light gray), systemic metastases (gray), and brain metastases (dark gray) of known BRAF/NRAS mutation status, and therapeutic record are summarized.

**Fig. 2:**
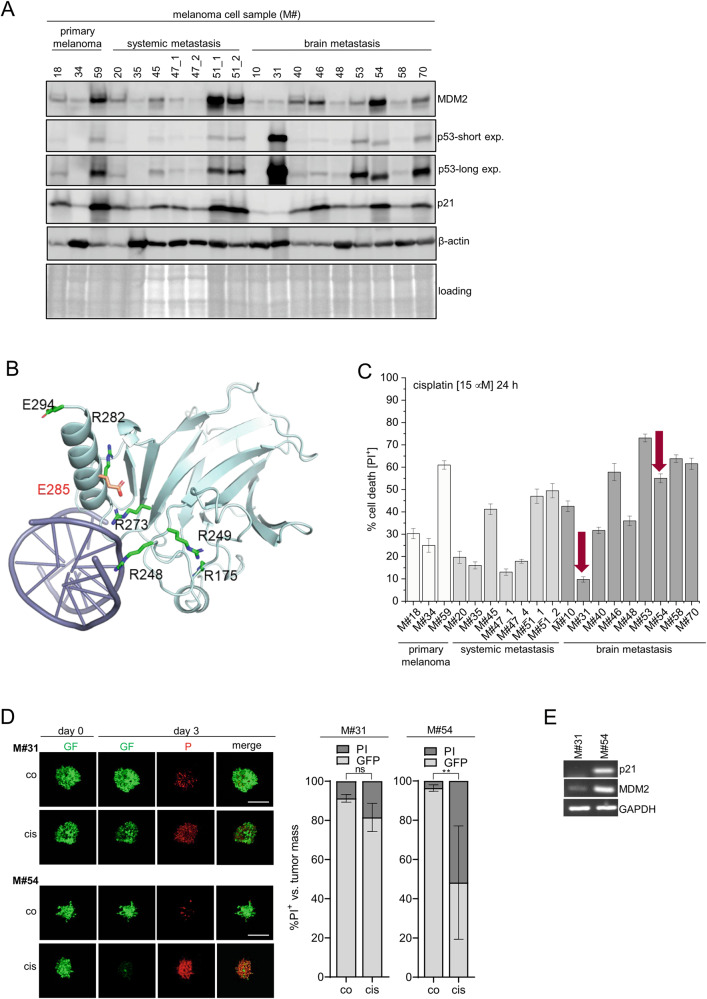
p53-E285K mutation is localized close to the DNA-binding domain and renders melanoma cells resistant to cisplatin-induced cell death. **A** Expression of p53, MDM2, and p21 of unstimulated melanoma cell samples was assessed by Western-blot analysis with β-actin and Ponceau S staining as loading controls. **B** The DBD domain of p53 bound to DNA: The designated hotspot mutations of p53 are shown in green. E285 residue is depicted in red. **C** Cell death induction (PI^+^) was monitored 24 h after stimulation of MM cell samples with cisplatin (15 µM) (*n* = 3; mean ± SD). Red arrows point at cell death responses of *mut*p53(E285K)-expressing M#31 and *wt*p53-expressing M#54 cells. **D** Individual GFP-expressing M#31 and M#54 spheroids were stimulated with cisplatin (15 µM) for 72 h. Cell death was visualized upon addition of PI (6.7 µg/ml). Confocal images of individual spheroids at day one and three as well as the quantification of tumor volume (green) *versus* tumor death (red) at day three are shown (*n* = 3; mean ± SD; **p ≤ 0.01; ns not significant). **E** Transcription of p21 and MDM2 in M#31 and M#54 cells, respectively, was assessed by RT-PCR. GAPDH served as housekeeping expression control.

To this end, our patient cohort convincingly represented the frequency (21.05%) and the heterogeneity of p53 mutations in MM. However, the p53 mutation status neither correlated with the BRAF/NRAS mutation status nor with MM localization (host tissue) or therapeutic stage, indicating that p53 mutation happens independently of these parameters. Moreover, six out of the remaining 15 MM samples expressed the p53 P72R point mutation (c.215C > G) considered to be a phenotypically silent single nucleotide polymorphism (SNP) [43]. Similar to M#10, M#34 and M#35 did not express the p53 protein, and hence p53 transcription-dependent MDM2 and p21 proteins remained absent in these loss-of-function variants (Fig. 2A). *Mut*p53(E285K) expression in M#31 appeared to be the strongest amongst all MM samples but also lacked MDM2 and p21 expression implying that *mut*p53(E285K) is not transcriptionally active [15, 16]. The structure of *wt*p53 bound to DNA suggested E285K mutation to be localized in a C-terminal helix of the DBD, which is in close proximity but not part of the DNA recognition site (Fig. 2B). More detailed structural analysis using Rosetta software [38] revealed E285K substitution to reduce conformational stability of the DBD, thereby putatively interfering with its function. Similar impact of E285K substitution was predicted by Polyphen2 [44], SIFT [45], and REVEL [46] tools (Table S3).

Responses of all MM samples to targeted *mut*BRAF inhibitor dabrafenib, downstream MEK1 inhibitor trametinib, or the clinically relevant co-inhibition remained very low, 10–20%, irrespective of the intrinsic BRAF/NRAS or p53 mutation status (Fig. S1). Upon treatment with the chemotherapeutic drug cisplatin, however, *wt*p53-expressing M#59, M#51_1/M51_2, M#46, M#48, M#53, M#54, M#58, and M#70 samples responded with 45–75% cell death, followed by p53(P72R-SNP)-expressing M#18, M#20, M#45, M#47_1/47_4, M#40 and M#48 cells (~50%). Surprisingly, M#10 cells featuring genomic p53 loss, remained quite sensitive to cisplatin-induced cell death (40%), whereas somatic p53 loss in M#34 and M#35 reduced cell death to 25–15%. M#31 stayed most resistant to cisplatin treatment (˂10%), suggesting *mut*p53(E285K) to serve a loss- or gain-of-function (Fig. 2C). To understand the role of *mut*p53(E285K) in cisplatin resistance we examined M#31 brain metastasis along with *wt*p53-expressing brain metastasis M#54. Cisplatin resistance of M#31 was confirmed comparing GFP-expressing M#31 and M#54 spheroids in an in vivo-mimicking 3D setting [30]. Three days after continuous cisplatin treatment, quantification of tumor mass (green) *versus* % PI-positive (PI^+^, red) revealed significant cell death induction in M#54 spheroids but had only marginal effects on M#31 spheroid survival (Fig. 2D). Finally, RT-PCR confirmed expression of p53-responsive p21 and MDM2 genes to remain absent in *mut*p53(E285K)-expressing M#31 cells, while being present in *wt*p53-expressing M#54 cells (Fig. 2E). Conclusively, E285K missense mutation appears to interfere with DNA-binding.

### Cytosolic localization of *mut*p53(E285K) protein is independent of K285 ubiquitination

**Fig. 3:**
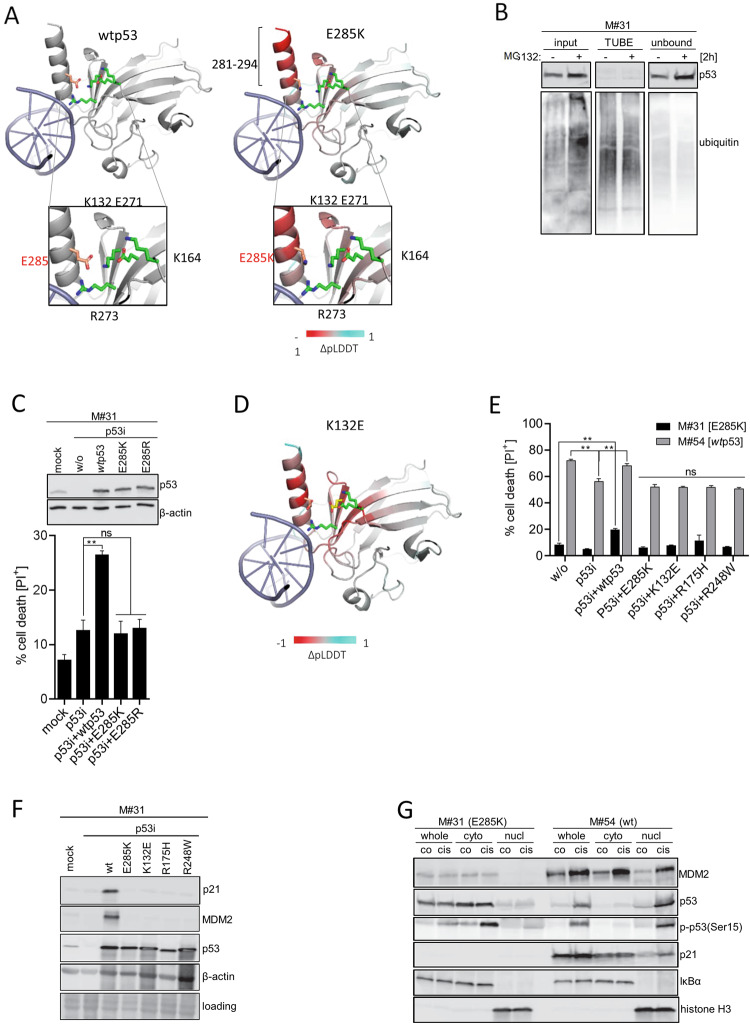
Cytosolic expression of *mut*p53(E285K) protein is independent of putative K285 ubiquitination. **A** The effect of the E285K mutation on 281–294 helix stability was validated compared to *wt*p53 using ESMFold model. Red color indicates reduction in protein stability according to changes in pLDDT score from red (destabilizing) to cyan (stabilizing). *Mut*p53(E285K) is shown in red. Magnifications illustrate the molecular interaction network involving E285 (left) *versus* K285 (right). The key residues involved in this network: K132, E271, K164, and R273 are shown in green. The structure is colored according to changes in protein stability due to mutation as estimated by the ESMFold ΔpLDDT score from red (destabilizing) to cyan (stabilizing). **B** M#31 cells were pretreated or not with MG132 (10 µM) for 2 h and a TUBE assay performed. **C** M#31 cells silenced for endogens *mut*p53(E285K) were reconstituted with *w*tp53, *mut*p53(E285K), and *mut*p53(E285R), respectively, and cell death (PI^+^) monitored 24 h after stimulation with cisplatin (15 µM) using IncuCyte® technology (*n* = 3; mean ± SD; **p ≤ 0.01; ns not significant). **D** Prediction of the effect of K132E mutation on the p53 DBD structure using ESMFold model. The structure is colored according to changes in protein stability due to mutation as estimated by the ESMFold ΔpLDDT score from red (destabilizing) to cyan (stabilizing). K132E and E285K mutations are depicted in yellow and red, respectively. The other residues involved in the salt-bridge network formation (K132, E271, K164, and R273) are shown in green. **E** M#31 and M#54 cells stably silenced for endogenous p53 were reconstituted with *wt*p53, and *mut*p53 variants E285K, K132E, R175H, and R248W, respectively, and cell death (PI^+^) monitored 24 h after stimulation with cisplatin (15 µM) (*n* = 3; mean ± SD; **p ≤ 0.01; ns not significant). **F** The same M#31 transfectants were used to assess p21 and MDM2 expression by Western-blot analysis. β-actin served as loading control. **G** M#31 (E285K) and M#54 (*wt*) cells were stimulated with cisplatin (15 µM). After 24 h localization of MDM2, p53, p-p53(Ser15), and p21 was monitored in whole cell lysates as well as in cytosolic and nuclear fractions by Western-blot analysis. IκBα and histone H3 served as loading/fractionation controls.

According to the p53 structure, E285 takes part in formation of a salt-bridge network comprising electrostatic interactions of positively and negatively charged amino acid residues: E285, K132, E271, K164, and R273 (Fig. 3A). The amino acid exchange at position 285 from E to K causes a switch of charges leading to structural destabilization of this salt-bridge network entailing conformational changes of the p53 protein. For validation we computed the structural models of *wt*p53 and *mut*p53(E285K) using ESMFold [36], allowing to predict the tertiary structure of a target protein from amino acid sequence information alone, and providing the model quality metrics (pLDDT) for each amino acid residue. Indeed, ΔpLDDT analysis suggested E285K substitution to decrease stability of the aa281-294 region (Fig. 3A**;** Table S4), thereby affecting stability of the DBD itself (Table S3), and the area that connects the DBD to the tetramerization domain (TET) (compare Fig. 1C). Performing native-PAGE revealed complex formation of *mut*p53(E285K) in response to cisplatin treatment not to be altered compared to *wt*p53 (Fig. S2), implying that not tetramerization but rather DNA recognition is predominantly affected through destabilization of the salt-bridge network. Importantly, the salt-bridge network includes one hotspot for p53 gain-of-function mutation (R273), which directly interacts with DNA, and may be affected by structural changes due to E285K mutation thereby potentially serving an indirect gain-of-function.

To test if exposure of K285 within this less stable p53 protein conformation relies on K285 ubiquitination we performed TUBE assay. Addition of the proteasome inhibitor MG132 clearly caused overall accumulation of ubiquitinated proteins in the input lysates, however, *mut*p53(E285K) did not become ubiquitinated itself and, thus, remained unbound to the TUBE (Fig. 3B). In contrast, ubiquitinated *wt*p53 in M#54 cells became strongly accumulated and upon proteasomal inhibition and, hence, was captured by TUBE pull down (Fig. S3). These data imply that the switch of charge at position 285 alone is sufficient to cause conformational changes leading to a complete loss-of-function phenotype in *mut*p53(E285K) expressing cells. To confirm this concept, we reconstituted M#31 cells stably silenced for endogenous p53 with *wt*p53, as well as with E285K and a E285R p53 variant, which also provides the positive charge but is incapable of being ubiquitinated. Only *wt*p53 re-expressing M#31 cells responded to cisplatin with significant cell death induction, whereas both, *mut*p53(E285K) and *mut*p53(E285R) re-expression had no effect (Fig. 3C), confirming that destabilization of the salt-bridge network - independent of ubiquitination - is sufficient to cause cisplatin resistance in M#31 cells. Since E285 is in direct contact with K132 within this salt-bridge network (Fig. 3D), RosettaDDG software and ESMFold predicted K132E conversion to have a similar functional effect as E285K substitution (Fig. 3D; Table S5).

Accordingly, we investigated the effect of E285K along with K132E mutation in comparison to designated R175H and R248W gain-of-function p53 mutants on cisplatin-induced cell death in M#31 and M#54 cells stably silenced for endogenous p53. Intriguingly, silencing of *wt*p53 in M#54 only moderately but significantly reduced cisplatin-induced cell death, and was fully restored by re-expression of *wt*p53, implying that the presence and/or mutation status of p53 may not exclusively decide about cellular fate in response to DNA damage induction (Fig. 3E; Fig. S4A, B). Reconstitution of either E285K or K132E - just like R175H and R248W gain-of-function mutants - reduced cell death responses to the level of p53-silenced M#54 cells. As predicted, none of the p53 mutants but only ectopic expression of *wt*p53 slightly increased cell death in M#31 cells in response to cisplatin, supporting that affecting the salt-bridge network may phenotypically cause loss of p53 function. This becomes even more evident by showing that exclusively reconstitution of *wt*p53 but neither of E285K and K132E p53 variants, nor R175H and R248W gain-of-function p53 mutants re-gained p21 and MDM2 expression in M#31 cells (Fig. 3F). Following this line, only reconstitution of M#10 patient samples harboring genomic loss of p53 as well as M#34 samples presenting with somatic p53 loss with *wt*p53 but not with *mut*p53(E285K) re-gained expression of p21 and MDM2 RNA and proteins (Fig. S5A–D). To this end, data allow to conclude that E285 missense mutation causes complete failure of p53 to recognize responsive promoter elements.

Intriguingly, we found *mut*p53(E285K) to be exclusively localized in the cytosol of M#31 cells (Fig. 3G), with no significant recruitment to mitochondria (Fig. S6). While nuclear *wt*p53 in M#54 cells becomes upregulated and phosphorylated at Ser15 in response to cisplatin, cytosolic *mut*p53(E285K) also becomes phosphorylated but only a small fraction translocates into the nucleus, certainly contributing to the lack of transcriptional activity (MDM2, p21) of *mut*p53(E285K).

### Mutations within the E285 cluster show high prevalence in melanoma and other cancer types

**Fig. 4:**
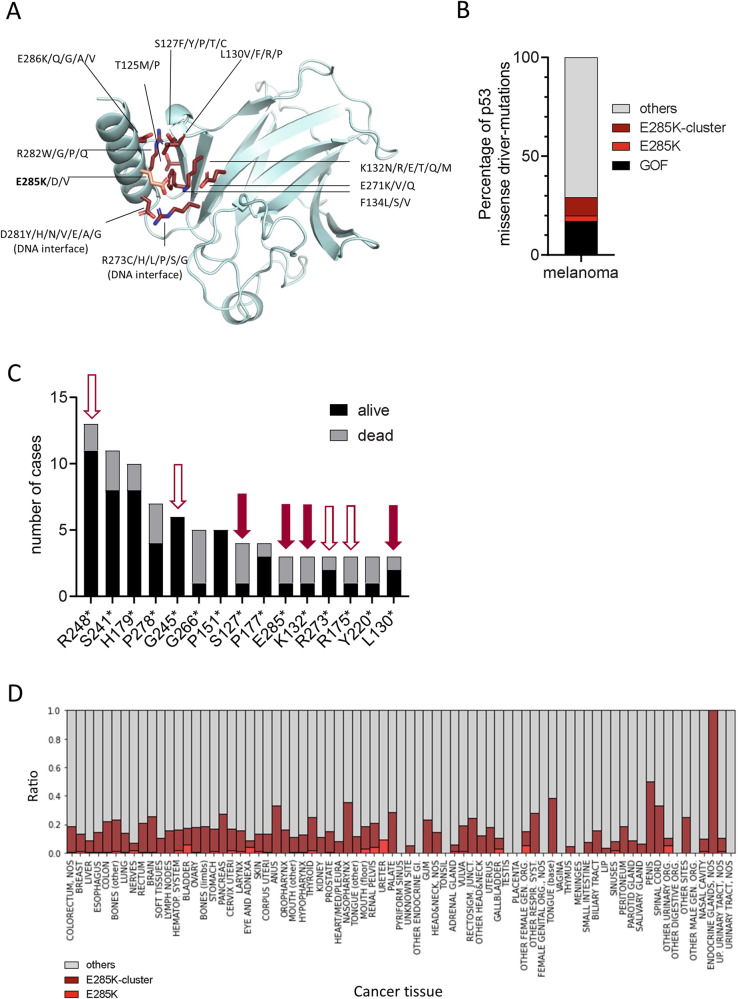
Mutations within the E285 cluster show high prevalence in melanoma and other cancer types. **A** The cluster of amino acid mutations within p53 that can have a similar functional effect on the C-terminal helix of the DBD domain as E285K. The E285K mutation is shown in light red, all other mutations in dark red. Mutant residues forming contacts interacting with DNA in the DNA-bound p53 complex are indicated (“DNA interface”). **B** Prevalence of E285-cluster mutations and in particular E258K mutation compared to other p53 mutation variants in MM, and **C** the frequency of most common p53 mutation in MM was correlated with the patient outcome (alive vs death) https://www.cbioportal.org/study/summary?id=msk_met_2021. Filled red arrows point at mutations being present in the E285 cluster, open red arrows at designated gain-of-function mutations. **D** The frequency of p53 missense mutations in various cancer tissues. The ratio of E285* (light red), of mutations within the E285 cluster (dark red), and all other p53 mutations (gray) as derived from https://tp53.isb-cgc.org is shown.

A more detailed model-based analysis revealed a whole cluster of amino acids frequently being mutated in p53-expressing cancers that may have a similar functional effect on the C-terminal helix of the DBD domain as E285K (E285 cluster) (Fig. 4A). Database analysis at https://www.cbioportal.org/study/summary?id=msk_met_2021 [31] revealed that the percentage of mutations within the E285 cluster (12%) is almost as high as of gain-of-function mutations at positions R175*, R248* and R273* (18%) (Fig. 4B). In particular, MM patients carrying missense mutations at positions S127*, E285*, and K132* within the E285 cluster present with elevated death rates comparable to patients with R175* or R273*, but clearly higher than R248* or G245* hotspot gain-of-function p53 mutations (Fig. 4C). Intriguingly, mutations within the E285 cluster and particularly E285K showed a high prevalence not only in MM but in a wide range of other cancer types, especially in those of endocrine glands (https://tp53.isb-cgc.org; [34]; Fig. 4D). Providing this global and pronounced prevalence of E285-cluster mutations, we aimed to identify a strategy to sensitize *mut*p53(E285K)-expressing cancer cells to chemotherapy.

### *Mut*p53(E285K) gain-of-function expressing M#31 cells can be sensitized to cisplatin using PRIMA1-Met and AKT inhibition, respectively

**Fig. 5:**
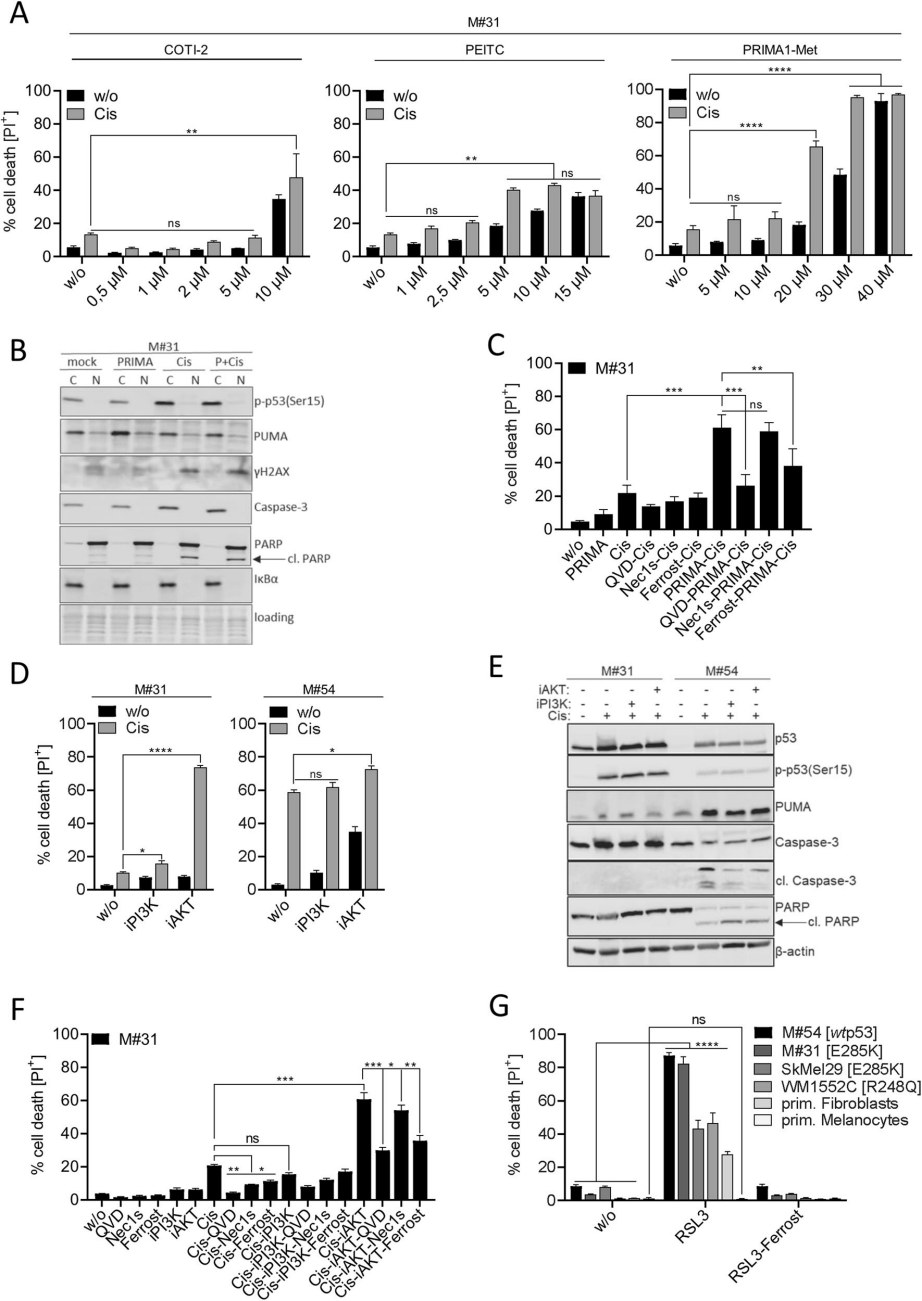
*Mut*p53(E285K) gain-of-function expressing M#31 cells can be sensitized to cisplatin using PRIMA1-Met and AKT inhibition, respectively. **A** M#31 cells were pretreated for 1 h with COTI-2, PEITC, and PRIMA1-Met at the indicated concentrations and cell death (PI^+^) monitored 24 h after stimulation with cisplatin (15 µM) (*n* = 3; mean ± SD; ****p ≤ 0.0001; **p ≤ 0.01; ns not significant). **B** The effect of PRIMA1-Met treatment on the subcellular localization and expression of p53, p-p53(Ser15), γH2AX, Caspase-3, PUMA, and PARP was monitored in cytosolic/nuclear fractions by Western-blot analysis. Ponceau S staining and IκBα served as loading/fractionation controls. **C** M#31 cells were pre-stimulated with QVD (5 µM), Nec1s (15 µM), or ferrostatin-1 (15 µM) for 1 h followed by treatment with PRIMA1-Met (20 µM). 24 h after cisplatin (15 µM) treatment cell death (PI^+^) was monitored (*n* = 3; mean ± SD; ***p ≤ 0.001; **p ≤ 0.01; ns not significant). **D** M#31 and M#54 cells were pretreated with alpelisip (iPI3K, 10 µM) and afuresertib (iAKT, 10 µM), respectively, for 1 h and cell death (PI^+^) in response to cisplatin (15 µM) determined after 24 h (*n* = 3; mean ± SD; ****p ≤ 0.0001; *p ≤ 0.05; ns not significant). **E** Changes in expression of p53, p-p53(Ser15), PUMA, Caspase-3, cleaved Caspase-3 and PARP was assessed by Western-blot analysis with β-actin as loading control. **F** M#31 cells were pre-stimulated with QVD (5 µM), Nec1s (15 µM), or ferrostatin-1 (15 µM) for 1 h followed by treatment with either alpelisip (iPI3K, 10 µM) or afuresertib (iAKT, 10 µM), and cell death (PI^+^) monitored after 24 h (*n* = 3; mean ± SD; ***p ≤ 0.001; **p ≤ 0.01; *p ≤ 0.05; ns not significant). **G** M#31 and M#54 cells, SkMel29 and WNM1552C MM cell lines, and primary human fibroblasts and melanocytes were pretreated or not with ferrostatin-1 (15 µM) for 1 h and cell death (PI^+^) in response to RSL3 (1.13 µM) determined after 24 h (*n* = 3; mean ± SD; ****p ≤ 0.000; ns not significant).

Restoration of *wt*-function of *mut*p53 in human cancers has been a task for many years, however, most (pre-)clinical studies have been performed using p53 hotspot mutants [16]. We investigated p53-dependent (re-)sensitization of *mut*p53(E285K)-expressing M#31 cells to cisplatin-induced cell death using three compounds, COTI-2, PEITC, and PRIMA1-Met which have been included into multiple clinical trials (https://www.cancer.gov/about-cancer/treatment/clinical-trials/intervention/mutant-p53-activator-coti-2; https://clinicaltrials.gov/ct2/show/NCT00691132; https://aacrjournals.org/mct/article/12/11/2331/91591/PRIMA-1Met-APR-246-Displays-High-Antitumor; [16]). Low doses of COTI-2 and PEITC had only marginal effects on cisplatin-induced cell death, but displayed cisplatin-independent cytotoxicity at higher doses (Fig. 5A). Treatment with PRIMA1-Met instead pronouncedly enhanced cisplatin-induced cell death, with highest synergy at 20 µM (Fig. 5A). As expected, none of the respective drugs further enhanced cisplatin-induced cell death in *wt*p53-expressing M#54 cells (Fig. S7). In M#31 cells, however, sensitization to cisplatin by PRIMA1-Met co-treatment neither enhanced nuclear translocation of *mut*p53(E285K) compared to cisplatin alone, nor did it cause p53-dependent upregulation of pro-apoptotic PUMA (Fig. 5B). Hence, caspase-3 processing and pronounced PARP cleavage, being indicative for apoptotic cell death, remained absent (Fig. 5B), questioning whether restoration of p53 *wt*-function is the primary mode of action of PRIMA1-Met. Accordingly, cell death in response to co-treatment with PRIMA1-Met and cisplatin could partially be rescued by inhibitors for apoptosis (QVD), and also of ferroptosis (ferrostatin-1), but not necroptosis (Nec1s), indicating a mixed form of apoptotic and ferroptotic cell death to be induced (Fig. 5C).

An alternative strategy to enhance cell death in MM is concomitant inhibition of MAPK-dependent survival pathways [47]. Since inhibition of the MEK-dependent branch of MAPK signaling proved ineffective in MM samples (compare Fig. S1), we selected clinically relevant alpelisip (iPI3K) and afuresertib (iAKT) to inhibit PI3K and AKT signaling [30], respectively. Only iAKT but not iPI3K synergistically sensitized *mut*p53(E285K)-expressing M#31 cells to cisplatin-induced cell death (70%) without showing cisplatin-independent cytotoxicity. In contrast, iAKT showed some toxicity in *wt*p53-expressing M#54 cells, but neither iAKT nor iPI3K pronouncedly enhanced cisplatin-dependent cell death (Fig. 5D). However, apoptosis induction was only evident in M#54 cells, due to upregulation and phosphorylation of *wt*p53, followed by p53-driven upregulation of PUMA and processing of caspase-3 and PARP, respectively. (Fig. 5E). In M#31 cells, phosphorylation of p53 did not result in transcriptional upregulation of PUMA, and consecutive apoptotic features, and hence, could only be partially inhibited with QVD (Fig. 5F). Similar to co-treatment with PRIMA1-Met, co-treatment with iAKT and cisplatin could partially be rescued by ferrostatin-1, confirming that M#31 cells were prone to ferroptosis induction (Fig. 5F). Conclusively, application of RSL3 a specific inhibitor of GPX4, induced extensive ferroptotic cell death (~90%) in *mut*p53(E285K)-expressing M#31, but also in *wt*p53-expressing M#54 cells. To estimate the clinical potential of RSL3 in treating MM we additionally investigated *mut*p53(E285K)-expressing SkMel29 and *mutp*53(R248Q) gain-of-function expressing WM1552C MM cell lines (https://p53.fr/tp53-database/the-tp53-cell-line-compendium), both being largely resistant to cisplatin as well as to re-sensitization with PRIMA1-Met (Fig. S8). Both cell lines responded with ~40–50% cell death to RSL3 treatment which was superior to 25% cell death being induced in primary fibroblasts. Most importantly, primary melanocytes fully resisted ferroptosis induction.

Taken together, we have identified an important cluster of p53 mutations, referred to as E285 cluster, being located close to the DBD indirectly interfering with DNA recognition and transcription of p53 target genes, presenting with high prevalence in MM but also in a wide range of other cancers. Our data furthermore challenge the concept of including ferroptosis-inducing agents into the treatment portfolio of MM, and most likely also for other cancer specimens.

## Discussion

To date, intervention strategies for the treatment of MM are exclusively based on the mutation status of the key drivers of metastatic progression, BRAF and NRAS oncogenes. Besides these genetic alterations other pathophysiological modifications, including the p53 mutation status, may contribute to therapy resistance [48].

Hotspot gain-of-function mutants account for ~28% of all missense mutations in p53 and have been investigated intensively [49, 50]. Much less information is available on residual ~72% missense mutations, most of which are also located within or adjacent to the DNA-binding domain [51].

Investigating the p53 mutation status in 19 patient-derived melanoma samples we have identified one p53 missense mutation E285K, previously described as a rare missense mutation being endowed with temperature-sensitive properties. At the permissive temperature of 32 °C this mutant was shown to regain wild-type properties allowing for profiling of p53-responsive genes [26]. Our data show *mut*p53(E285K) to be localized in the cytosol of M#31 cells and to only partially translocate into the nucleus in response to DNA damage, thereby conferring extensive chemoresistance. Cytosolic p53 cluster have recently gained increased attention, because they were found to correlate with poor prognosis in serous ovarian carcinoma [52], and to couple oncogene-driven metabolism to apoptosis in glioblastoma [53]. Restoration of *mut*p53(E285K), in M#31 and M#54 cells silenced for endogenous p53, as well as in M#10 and M#34 lacking endogenous p53 protein, prevented p53-driven transcription of p21, MDM2, and PUMA, and reduced cisplatin responses to the level of gain-of-function R175H and R248W re-expressing cells. Co-treatment with PRIMA1-Met (re)sensitized M#31 to cisplatin, however, this appeared to be independent of restoration of p53 *wt*-function. Multiple clinical trials have been conducted incorporating PRIMA1-Met (APR-246), showing some promising results when co-administered with conventional drugs in several hematologic malignancies and solid tumors. Unfortunately, a study on melanoma treatment was discontinued in 2019 (reviewed in [16]). All three drugs used in this study aiming to restore p53 *wt*-function (COTI-2, PEITC, PRIMA1-Met) have proven cytotoxic effects on various cancer types beyond p53 reactivation, mostly due to reactive oxygen (ROS) formation inducing oxidative stress [18, 54–56], which may contribute to lipid peroxidation, and thus, to ferroptosis [57]. This may explain partial ferroptosis induction upon PRIMA1-Met and cisplatin co-treatment. Moreover, AKT was recently shown to inhibit GPX4 degradation through creatine kinase B-dependent phosphorylation, thereby mitigating ferroptosis in hepatocellular carcinoma cell lines [58]. This in turn may explain how AKT inhibition may contribute to ferroptosis induction. Since clinical trials involving PI3K [59] or AKT [60] inhibition, alone or in combination with conventional *mut*BRAF or MEK targeting drugs have shown poor benefit for MM patients, direct ferroptosis induction may provide a promising alternative. We show GPX4 inhibition by RSL3 to tumorselectively induce extensive ferroptosis in wild-type and mutant p53-expressing MM cells while sparing primary cells of the skin, implying ferroptosis induction to be a viable alternative treatment option for MM irrespective of the intrinsic p53 mutation status. Accordingly, increasing effort is currently undertaken to implement ferroptotic marker identification into diagnostic measures as well as therapeutic strategies for the treatment of MM [61–63].

Our structural analysis revealed E285K to be part of a cluster of frequently occurring missense mutations (E285 cluster) in MM, but also in a wide range of other cancers. As a future task, small molecules that interfere with the destabilization of the C-terminal helix of the p53 DBD may represent a viable strategy to rescue the negative impact of E285-cluster mutations, thereby paving the way for alternative therapeutic options to fight cancer.

### Reporting summary

Further information on research design is available in the Nature Research Reporting Summary linked to this article.

### Supplementary information

Reporting Summary

Supplementary figures

Supplementary tables

## Data Availability

The datasets used and analyzed during the course of the current study are available from the corresponding author upon reasonable request. Uncropped Western Blots are available as Supplementary Data.
