# Supplementary material for: A cytosolic mutp53(E285K) variant confers chemoresistance of malignant melanoma
Source: Cell Death Dis. 2023 Dec 14;14(12):831. doi: 10.1038/s41419-023-06360-4 (PMC10721616; doi:10.1038/s41419-023-06360-4)
Supplement: Supplementary file 1 — Reporting Summary [file 41419_2023_6360_MOESM1_ESM.pdf]

Table S1: Related to Figure 1A. Top 15 most prevalent solid cancers worldwide. The most prevalent cancer entities were derived from https://gco.iarc.fr/today database (2020), and Non-Hodgkin lymphoma and leukemia excluded as being non-solid tumors.

Rank	Cancer	New cases in 2020	% of all cancers
	All cancers	18,094,716	
1	Breast	2,261,419	12.5%
2	Lung	2,206,771	12.2%
3	Colorectal	1,931,590	10.7%
4	Prostate	1,414,259	7.8%
5	Stomach	1,089,103	6.0%
6	Liver	905,677	5.0%
7	Cervix uteri	604,127	3.3%
8	Oesophagus	604,100	3.3%
9	Thyroid	586,202	3.2%
10	Bladder	573,278	3.2%
11	Non-Hodgkin lymphoma	544,352	3.0%
12	Pancreas	495,773	2.7%
13	Leukemia	474,519	2.6%
14	Kidney	431,288	2.4%
15	Corpus uteri	417,367	2.3%
16	Lip, oral cavity	377,713	2.1%
17	Melanoma of skin	324,635	1.8%

Table S2: Related to Figure 1A. Incidence of p53 mutations.
The incidence of p53 mutations in “all cancers” compared to “melanoma” as derived from https://www.cbioportal.org.

A all cancers

Gene	# Mut	#	Profiled Samples	Freq
TP53	13367	12450	25775	48.30%
KRAS	5523	5446	25775	21.10%
APC	5441	3643	25775	14.10%
PIK3CA	4163	3581	25775	13.90%
TERT	3129	2845	25775	11.00%
ARID1A	3205	2530	25775	9.80%
KMT2D	3162	2263	25775	8.80%
PTEN	2339	1787	25775	6.90%
KMT2C	2065	1640	25775	6.40%
EGFR	1971	1630	25775	6.30%
BRAF	1611	1545	25775	6.00%
FAT1	2032	1480	25775	5.70%
ATM	1841	1457	25775	5.70%
CDKN2A	1481	1412	25775	5.50%
ZFHX3	1784	1318	23974	5.50%
PTPRT	1828	1427	25775	5.50%
NF1	1830	1403	25775	5.40%
KMT2B	1268	929	17602	5.30%
SMAD4	1463	1370	25775	5.30%
PTPRD	1692	1268	25775	4.90%

B melanoma

Gene	# Mut	#	Profiled Samples	Freq
TERT	856	701	1142	61.40%
BRAF	422	395	1142	34.60%
PTPRT	526	304	1142	26.60%
PREX2	294	189	782	24.20%
NRAS	277	272	1142	23.80%
NF1	398	272	1142	23.80%
GRIN2A	441	267	1142	23.40%
PTPRD	409	247	1142	21.60%
TP53	284	245	1142	21.50%
ROS1	365	243	1142	21.30%
PAK5	305	224	1142	19.60%
KMT2D	303	197	1142	17.30%
PIK3C2G	298	196	1142	17.20%
ERBB4	278	193	1142	16.90%
KMT2B	175	128	782	16.40%
EPHA7	244	172	1099	15.70%
TP63	231	176	1142	15.40%
CDKN2A	193	172	1142	15.10%
FAT1	222	167	1142	14.60%
KDR	205	157	1142	13.70%

Table S3: Related to Figure 2B and 3A. Impact of E285K and “E285-cluster” mutations on the p53 DBD.

The impact of mutations was estimated based on SIFT, Polyphen-2, and REVEL predictions and protein stability changes predicted by RosettaDDGPrediction software. Positive value of RosettaDDG corresponds to a decrease in stability.

Mutation	# of cases	Polyphen2	SIFT-Class	REVEL	Rosetta ddG
E286K	99	D	Damaging	0.949	8.43
E286G	22	D	Damaging	0.913	10.61
E286D	4	B	Damaging	0.765	5.55
E286Q	16	D	Damaging	0.905	3.58
E286V	7	D	Damaging	0.916	6.68
E286A	2	D	Damaging	0.913	7.8
**E285K**	**186**	**D**	**Damaging**	**0.91**	**3.88**
E285Q	8	D	Damaging	0.883	1.51
E285V	21	D	Damaging	0.936	2.51
E285G	5	D	Damaging	0.923	3.84
E285D	2	D	Tolerated	0.524	-0.22
E285A	1	D	Damaging	0.91	0.17
D281G	18	D	Damaging	0.974	11.07
D281E	55	D	Damaging	0.909	6.31
D281H	43	D	Damaging	0.961	9.18
D281Y	11	D	Damaging	0.957	9.84
D281V	5	D	Damaging	0.969	8.7
D281N	37	D	Damaging	0.943	10.35
D281A	6	D	Damaging	0.977	7.42
R273H	863	D	Damaging	0.868	4.03
R273C	718	D	Damaging	0.901	4.04
R273P	38	D	Damaging	0.932	17.3
R273L	155	D	Damaging	0.921	0.7
R273G	19	D	Damaging	0.902	8.52
R273S	21	D	Damaging	0.906	3.27
E271K	38	D	Damaging	0.935	15.78
E271G	5	D	Damaging	0.967	8.66
E271V	9	D	Damaging	0.972	7.91
E271D	6	D	Damaging	0.843	10.25
E271Q	6	D	Damaging	0.924	7.17
E271A	2	D	Damaging	0.95	5.57
F134L	29	D	Damaging	0.942	2.79
F134S	3	D	Damaging	0.942	8.44
F134C	9	D	Damaging	0.949	9.34
F134I	2	D	Damaging	0.955	35.67
F134V	12	D	Damaging	0.959	15.67
K132Q	19	D	Damaging	0.944	1.71
K132N	74	D	Damaging	0.895	6.27
K132R	66	D	Damaging	0.908	4.74
K132E	25	D	Damaging	0.968	5.03
K132M	15	D	Damaging	0.924	1.19
K132T	6	D	Damaging	0.927	1.83
L130V	24	D	Damaging	0.887	5.92
L130R	11	D	Damaging	0.918	9.11
L130F	17	D	Damaging	0.921	12.15
L130I	2	D	Damaging	0.795	6.39
L130P	6	D	Damaging	0.927	26.14
L130H	6	D	Damaging	0.924	12.12
S127F	37	D	Damaging	0.925	9.15
S127P	8	D	Damaging	0.957	17.85
S127Y	12	D	Damaging	0.918	9.47
S127T	4	D	Damaging	0.924	3.21
S127C	2	D	Damaging	0.916	4.99
T125M	12	D	Damaging	0.925	2.56
T125K	4	D	Damaging	0.963	29.91
T125R	2	D	Damaging	0.963	26.97
T125P	1	D	Damaging	0.945	33.21
T125A	1	D	Damaging	0.943	2.65

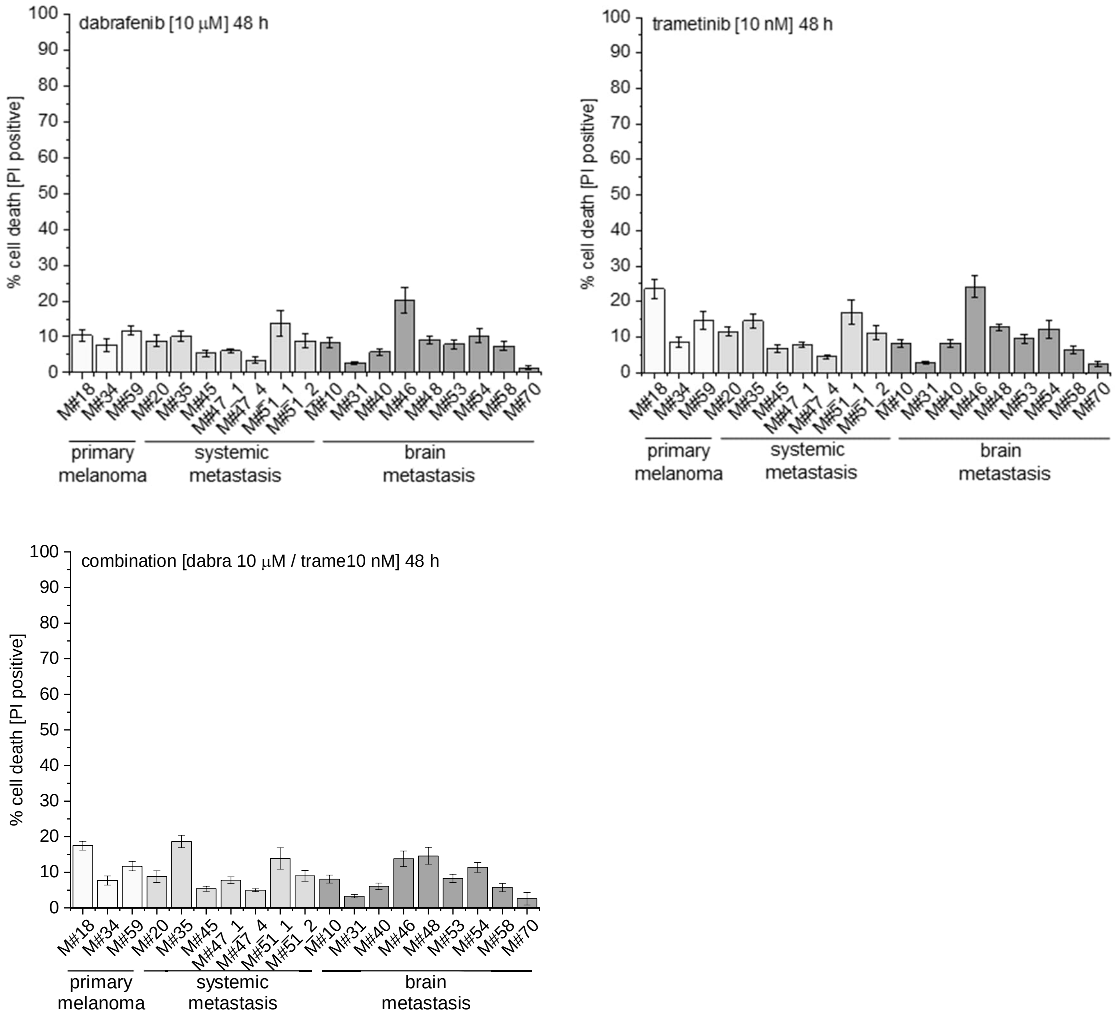

Fig. S1: Related to Figure 2C. Primary melanoma cell samples stay largely resistant against targeted *mut*BRAF and MEK inhibition. Cell death induction (PI^+) was monitored 48 h after stimulation of primary melanoma cell samples with *mut*BRAF inhibitor dabrafenib (10 µM), and MEK inhibitor trametinib (10 nM) individually, or in combintaton using IncuCyte® technology (n=3; mean ± SD).

Table S4: Related to Figure 3A. *In silico* prediction of the changes in stability of p53 DBD by E285K mutation.

The changes in p53stability were evaluated using PoPMusic , RosettaDDG, Maestro, PremPS, and ESMFold software. In the case of ESMFold differences in the pLDDT score of introducing mutations in *wt*- and *mut*- protein structures was considered.

Instrument /Computational tool	Stability changes prediction by E285K mutation
PoPMuSiC	0.66
Rosetta	3.87
Maestro	1.636
PremPS	0.87
ESMFold	3.82

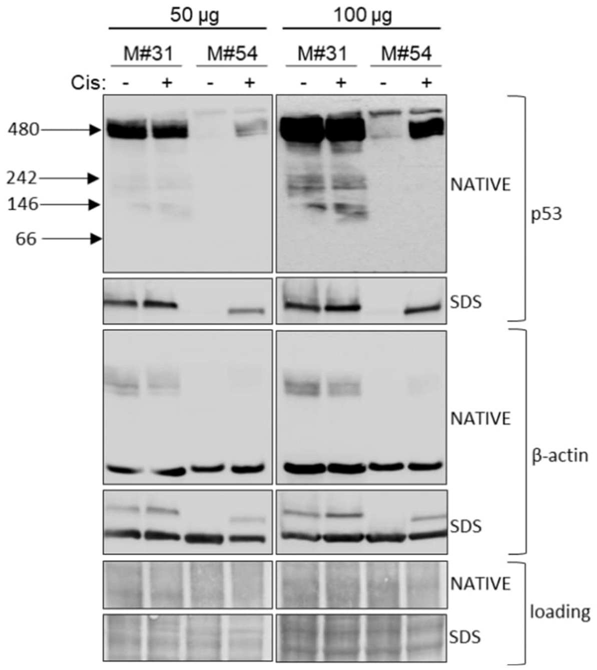

Fig. S2: Related to Figure 3A. Complex formation of *mut*p53(E285K) is not impaired compared to *wt*p53. *mut*p53(E285K)-expressing M#31 cells and *wt*p53-expressing M#54 cells were stimulated with cisplatin (15 µM) for 24 h. Subsequently 50 and 100 µg of native (NATIVE) and denaturated (SDS) protein extracts were generated and the p53 complexes assessed by NATIVE *versus* SDS-driven Western-blot analysis. β-actin and Ponceau-staining served as loading controls

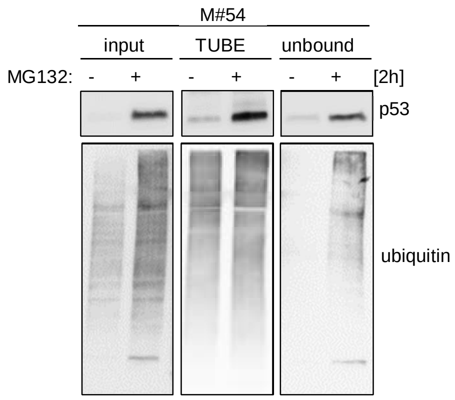

Fig. S3: Related to Figure 3B: TUBE assay using *wt*p53-expressing M#54 cell samples. *wt*p53-expressing M#54 cells were pretreated or not with MG132 (10 µM) for 2 h and a TUBE assay performed.

Table S5: Related to Figure 3D. **ESMFold p-LDDT scores for wild-type, E285K, K132E and E285K/K132E p53 protein models.**

Residue number	Wild-type amino acid	ESMFold pLDDT			
		Wilde-type	E285K	K132E	E285K/K132E
106	S	62.68	62.95	62.59	62.72
107	Y	80.96	81.17	81.1	81.12
108	G	86.4	86.53	86.47	86.48
109	F	89.2	89.3	89.29	89.26
110	R	78.86	78.95	78.91	78.91
111	L	90.79	90.93	90.72	90.78
112	G	91.78	91.93	91.72	91.78
113	F	90.04	90.23	89.9	90.03
114	L	78.64	79.13	78.62	78.87
115	H	68.02	69.08	68.15	68.67
116	S	79.83	80.49	79.94	80.38
117	G	85.83	86.32	86.11	86.44
118	T	83.53	84.78	84.39	84.9
119	A	87.46	87.94	87.79	88.01
120	K	75.56	76.2	76.08	76.29
121	S	86.44	86.88	86.78	86.93
122	V	85.37	85.61	85.58	85.68
123	T	91.77	91.92	91.79	91.81
124	C	93.74	93.85	93.63	93.76
125	T	92.67	92.7	92.5	92.63
126	Y	91.42	91.44	91.2	91.32
127	S	92.71	92.61	92.45	92.52
128	P	90.09	89.95	89.87	89.88
129	A	91.05	90.77	90.66	90.66
130	L	92.08	91.5	91.43	91.4
131	N	91.36	91.22	91.26	91.32
132	K	92.99	92.12	92.48	92.85
133	M	93.18	93.34	93.0	93.21
134	F	94.35	94.27	94.04	94.12
135	C	95.11	95.13	94.94	95.05
136	Q	92.13	92.02	92.07	92.12
137	L	90.39	90.4	90.29	90.25
138	A	91.57	91.6	91.53	91.53
139	K	88.65	88.75	88.58	88.62
140	T	90.02	90.15	90.08	90.12
141	C	92.68	92.8	92.61	92.66
142	P	92.31	92.43	92.33	92.38
143	V	93.73	93.83	93.72	93.76
144	Q	89.1	89.27	89.23	89.26
145	L	85.83	85.92	85.93	85.92
146	W	76.73	76.76	76.72	76.58
147	V	86.76	86.81	87.11	87.0
148	D	84.15	84.3	84.24	84.27
149	S	83.23	83.34	83.34	83.35
150	T	82.84	82.98	82.98	82.98
151	P	87.08	87.23	87.25	87.25
152	P	87.33	87.45	87.48	87.48
153	P	87.2	87.34	87.34	87.36
154	G	90.49	90.61	90.6	90.61
155	T	90.55	90.67	90.62	90.66
156	R	80.14	80.16	80.16	80.15
157	V	93.48	93.57	93.54	93.55
158	R	88.63	88.57	88.72	88.61
159	A	95.44	95.49	95.42	95.43
160	M	87.67	87.7	87.69	87.65
161	A	95.23	95.24	95.16	95.15
162	I	91.49	91.42	91.41	91.35
163	Y	92.89	92.83	92.69	92.68
164	K	88.67	88.4	87.24	87.38
165	Q	84.2	84.21	83.98	84.01

166	S	86.96	86.92	86.72	86.68
167	Q	81.93	81.95	81.85	81.76
168	H	88.28	88.3	88.24	88.17
169	M	79.76	79.79	79.73	79.65
170	T	84.52	84.53	84.5	84.4
171	E	87.45	87.51	87.38	87.33
172	V	90.91	90.96	90.92	90.88
173	V	93.84	93.85	93.82	93.78
174	R	77.76	77.72	77.84	77.8
175	R	85.47	85.51	85.5	85.42
176	C	91.61	91.61	91.64	91.61
177	P	88.45	88.47	88.5	88.47
178	H	81.25	81.24	81.28	81.27
179	H	89.6	89.64	89.61	89.6
180	E	84.61	84.62	84.67	84.57
181	R	74.35	74.48	74.42	74.46
182	C	75.1	75.29	75.16	75.26
183	S	55.66	55.68	55.61	55.62
184	D	60.68	60.72	60.74	60.73
185	S	54.16	54.22	54.18	54.17
186	D	56.13	56.25	56.18	56.18
187	G	66.43	66.58	66.49	66.44
188	L	71.58	71.66	71.69	71.66
189	A	83.5	83.6	83.63	83.59
190	P	86.14	86.18	86.26	86.19
191	P	85.6	85.61	85.7	85.63
192	Q	81.9	81.97	82.05	81.94
193	H	89.68	89.7	89.74	89.69
194	L	93.03	93.06	93.01	92.99
195	I	94.0	94.03	93.96	93.96
196	R	77.61	77.63	77.63	77.61
197	V	89.82	89.93	89.86	89.89
198	E	82.6	82.68	82.69	82.69
199	G	84.31	84.49	84.4	84.46
200	N	81.32	81.57	81.43	81.55
201	L	79.96	80.17	80.08	80.13
202	R	79.83	79.95	79.95	79.94
203	V	87.73	87.85	87.85	87.87
204	E	86.91	86.99	86.96	86.95
205	Y	89.17	89.21	89.22	89.21
206	L	88.28	88.34	88.32	88.27
207	D	88.95	88.99	89.04	88.97
208	D	86.1	86.24	86.17	86.16
209	R	74.17	74.28	74.29	74.24
210	N	84.48	84.61	84.54	84.56
211	T	87.75	87.84	87.83	87.8
212	F	86.27	86.31	86.34	86.29
213	R	84.27	84.28	84.34	84.29
214	H	88.91	88.92	89.1	89.01
215	S	92.67	92.72	92.71	92.69
216	V	89.39	89.49	89.38	89.4
217	V	92.31	92.41	92.35	92.37
218	V	92.06	92.19	92.09	92.13
219	P	92.02	92.12	92.1	92.12
220	Y	90.06	90.18	90.19	90.18
221	E	82.29	82.43	82.41	82.42
222	P	86.62	86.77	86.77	86.78
223	P	87.29	87.48	87.5	87.51
224	E	77.48	77.65	77.64	77.67
225	V	80.46	80.71	80.72	80.75
226	G	84.4	84.58	84.58	84.6
227	S	83.53	83.71	83.68	83.7
228	D	81.24	81.42	81.37	81.44
229	C	88.67	88.86	88.82	88.87
230	T	85.93	86.03	86.05	86.05
231	T	91.06	91.21	91.14	91.18
232	I	92.11	92.23	92.18	92.19
233	H	87.39	87.5	87.51	87.52
234	Y	92.99	93.08	92.99	93.02
235	N	85.03	85.14	85.14	85.2
236	Y	93.37	93.4	93.3	93.32
237	M	90.62	90.66	90.63	90.62
238	C	93.21	93.21	93.15	93.15
239	N	90.05	90.03	90.1	90.1
240	S	93.4	93.35	93.12	93.22

241	S	91.94	91.73	91.78	91.77
242	C	91.85	91.74	91.79	91.72
243	M	83.57	83.46	83.59	83.55
244	G	88.81	88.75	88.83	88.76
245	G	89.98	89.88	89.96	89.85
246	M	90.82	90.82	90.73	90.7
247	N	87.5	87.33	87.48	87.29
248	R	78.39	78.16	78.01	78.0
249	R	85.62	85.59	85.16	85.3
250	P	92.43	92.15	91.59	91.7
251	I	94.56	94.5	94.26	94.35
252	L	89.28	89.1	87.29	87.35
253	T	95.41	95.43	95.25	95.28
254	I	91.68	91.68	91.84	91.83
255	I	93.46	93.56	93.45	93.49
256	T	93.61	93.67	93.6	93.6
257	L	90.64	90.69	90.7	90.67
258	E	91.03	91.11	91.11	91.11
259	D	89.35	89.49	89.43	89.46
260	S	87.2	87.31	87.27	87.29
261	S	86.81	86.91	86.86	86.86
262	G	87.44	87.54	87.5	87.51
263	N	86.4	86.47	86.47	86.48
264	L	87.33	87.46	87.42	87.43
265	L	90.33	90.45	90.43	90.43
266	G	92.54	92.61	92.59	92.59
267	R	85.72	85.75	85.63	85.6
268	N	86.54	86.68	86.6	86.69
269	S	92.35	92.38	92.21	92.25
270	F	93.98	93.92	93.7	93.8
271	E	89.72	89.54	86.7	86.88
272	V	95.5	95.42	95.12	95.29
273	R	86.03	83.37	84.3	83.26
274	V	95.01	94.97	94.79	94.88
275	C	94.09	93.88	93.74	93.84
276	A	92.46	92.3	92.21	92.29
277	C	89.14	89.1	89.1	89.12
278	P	93.55	93.5	93.34	93.49
279	G	92.88	92.86	92.7	92.86
280	R	86.43	86.36	86.19	86.39
281	D	91.51	91.28	91.17	91.38
282	R	90.83	90.61	90.64	90.47
283	R	78.71	78.72	78.69	78.74
284	T	91.07	90.61	90.61	90.69
285	E	89.23	85.33	83.93	86.6
286	E	91.33	91.04	91.14	91.07
287	E	82.83	82.67	82.91	82.81
288	N	86.58	85.99	86.27	86.25
289	L	88.03	87.2	87.7	87.24
290	R	75.67	75.51	75.66	75.54
291	K	82.53	82.08	82.44	82.09
292	K	79.25	78.35	79.13	78.53
293	G	79.08	77.62	79.54	78.02

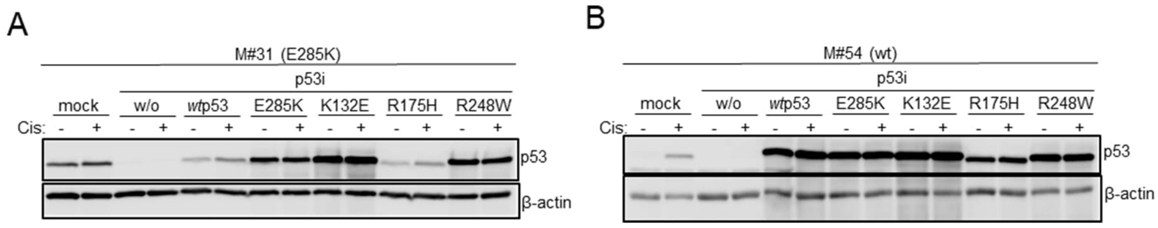

Fig. S4: Related to Figure 3E. Ectopic expression of p53 variants. A M#31 and **B** M#54 cells stably silenced for endogenous p53 were reconstituted with *wt*p53, and *mut*p53 variants E285K, K132E, R175H and R248W, respectively, and p53 expression assessed 24 h after stimulation with cisplatin (15 µM) by Western-blot analysis with β-actin as loading control. One representative out of three independently performed experiments is shown.

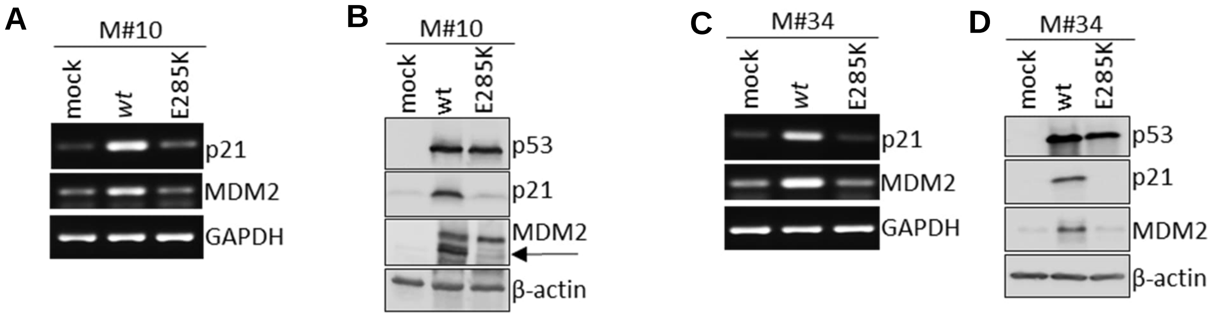

Fig. S5: Related to Figure 3E. Ectopic expression of *wt*p53 and *mut*p53(E285K) in cells lacking intrinsic p53 expression. A M#10 cells presenting with genomic loss of p53 and **C:** M#34 cells presenting with somatic loss of p53 were transfected with an empty vector or with *wt*p53 or *mut*p53(E285K)-expressing plasmids. 24 h later RNA was extracted and subjected to RT-PCR using primers directed against p21 and MDM2. GAPDH served as housekeeping expression control. **B:** p53, p21 and MDM2 expression of M#10 transfectants and **D:** of M#34 transfectants of was assessed by Western-blot analysis. β-actin served as loading control.

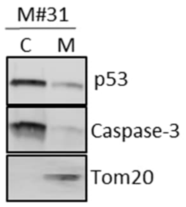

Fig. S6: Related to Figure 3F. Cytosolic *mut*p53(E285K) does not localize to mitochondria. Subcellular localization p53 was assessed by Western-blot analysis using cytosolic and mitochondrial fractions of M#31 cells. Caspase-3 served as a control for cytosolic, and Tom20 for mitochondrial localization. One representative out of three independently performed experiments is shown.

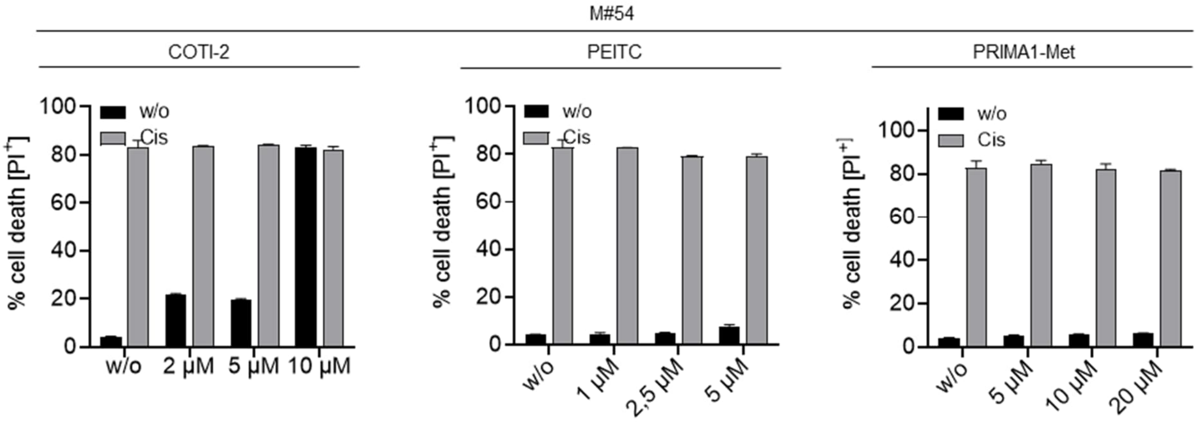

Fig. S7: Related to Figure 5A. Compounds restoring p53 *wt* function to not enhance cisplatin-induced cell death in M#54 cells. M#54 cells were pretreated for 1 h with COTI-2, PEITC and PRIMA1-Met at the indicated concentrations and cell death (PI+) monitored 24 h after stimulation with cisplatin (15 µM) using IncuCyte® technology (n=3, mean ± SD).

.

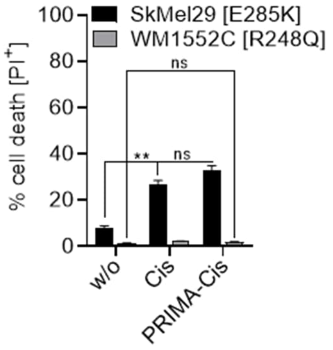

Fig. S8: Related to Figure 5G. *Mut*p53(E285K)-expressing SkMel29 and *mut*p53(R175H)-expressing MW1552C cells only slightly respond to cisplatin but not to PRIMA1-Met. SkMel29 and WM1552C cells were pretreated for 1 h with PRIMA1-Met (20 µM) and cell death (PI+) monitored 24 h after stimulation with cisplatin (15 µM) using IncuCyte® technology (n=3, mean ± SD **p ≤ 0.01; ns = not significant).
