# Supplementary material for: A cytosolic mutp53(E285K) variant confers chemoresistance of malignant melanoma
Source: Cell Death Dis. 2023 Dec 14;14(12):831. doi: 10.1038/s41419-023-06360-4 (PMC10721616; doi:10.1038/s41419-023-06360-4)

Figure 2A:

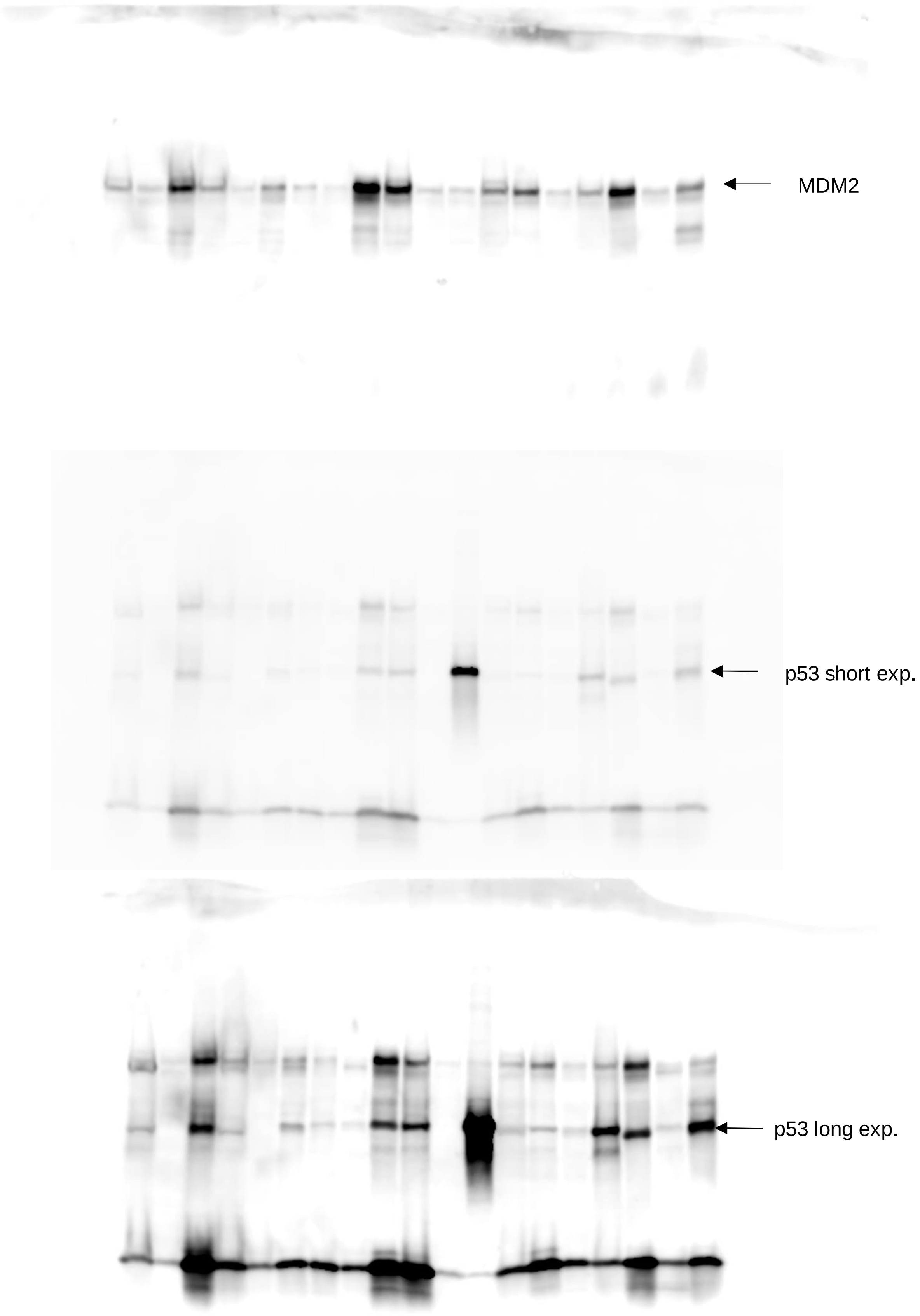

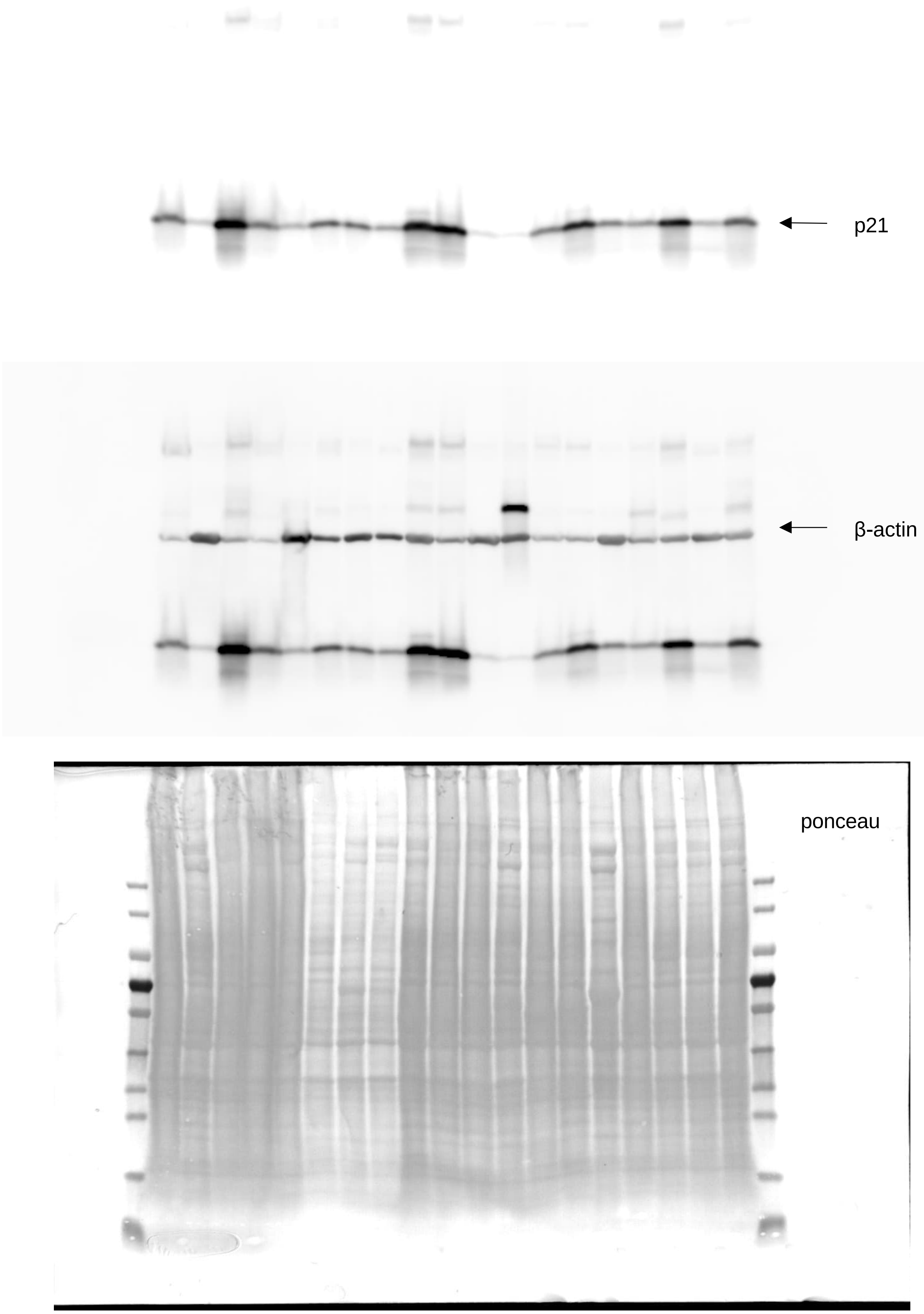
p21
β-actin
ponceau

Figure 3B:

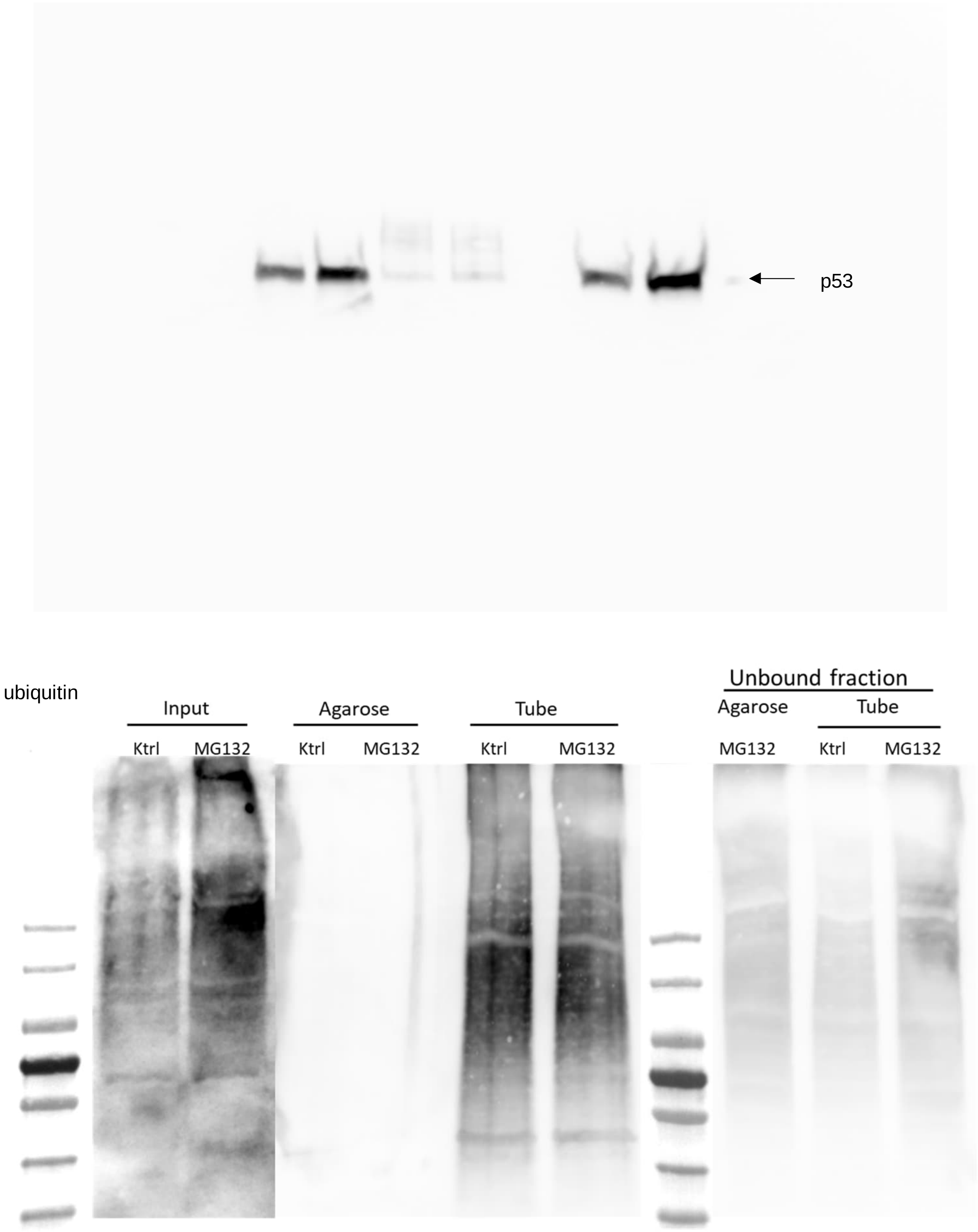

p53
ubiquitin
Input
Ktrl
MG132
Agarose
Ktrl
MG132
Tube
Ktrl
MG132
Unbound fraction
Agarose
MG132
Tube
Ktrl
MG132

Figure 3C:

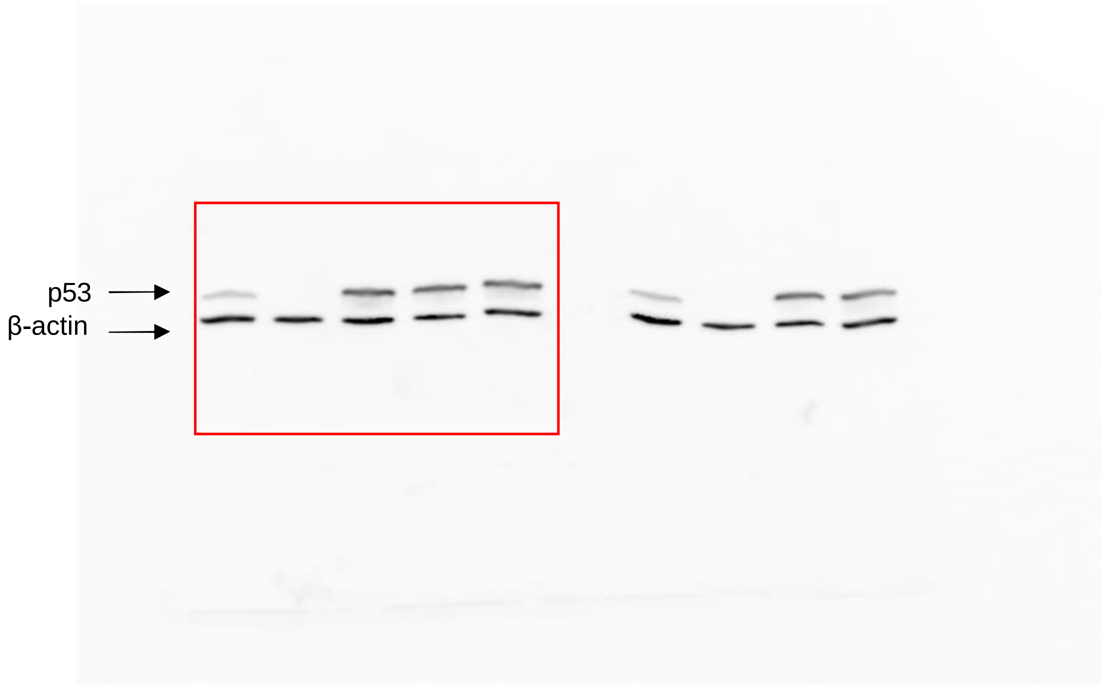

Figure 3F:

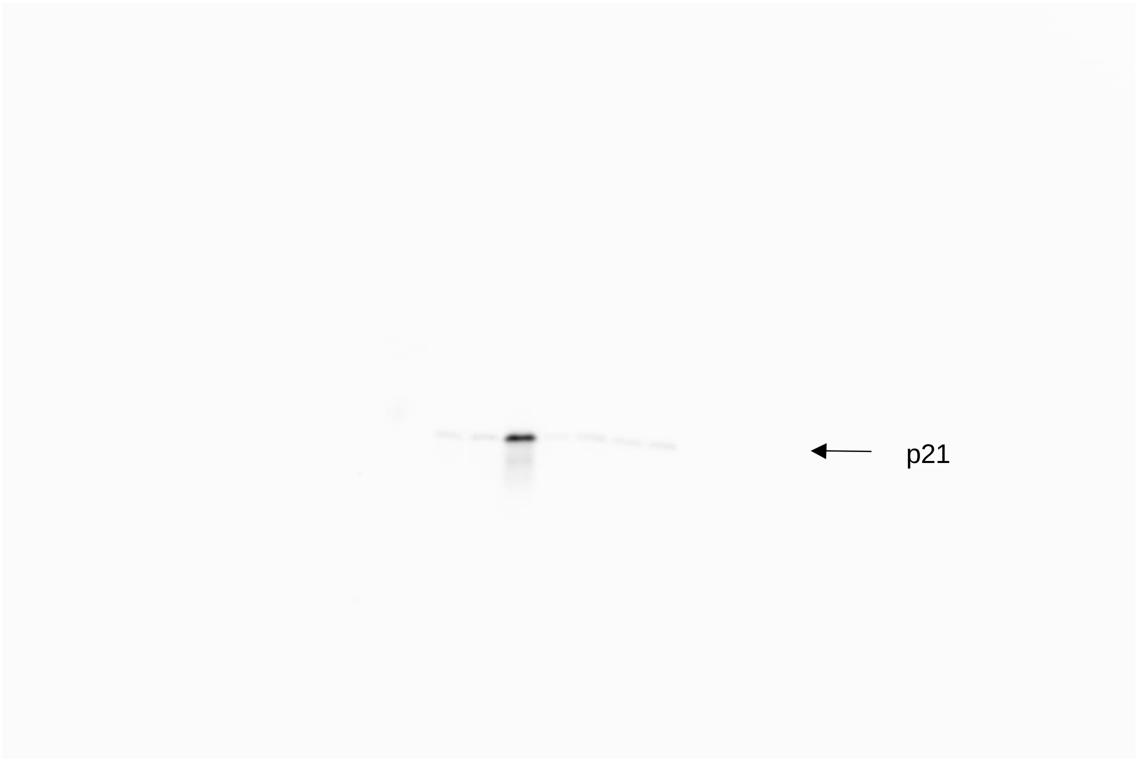

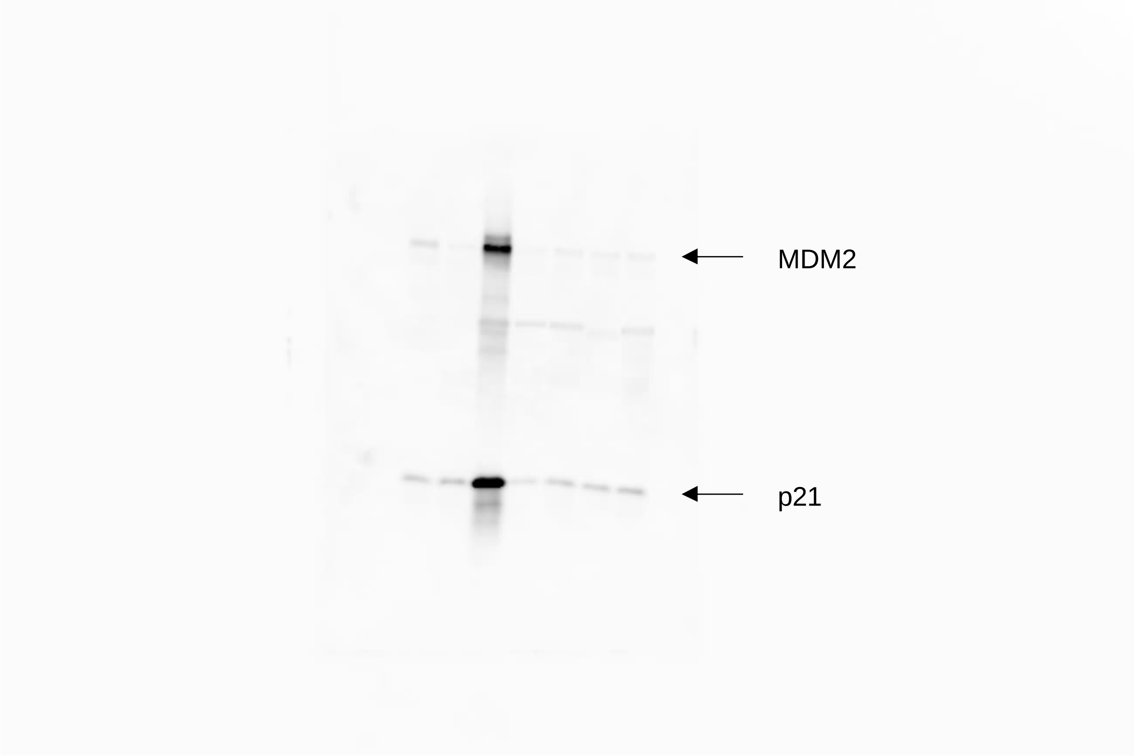
MDM2
p21

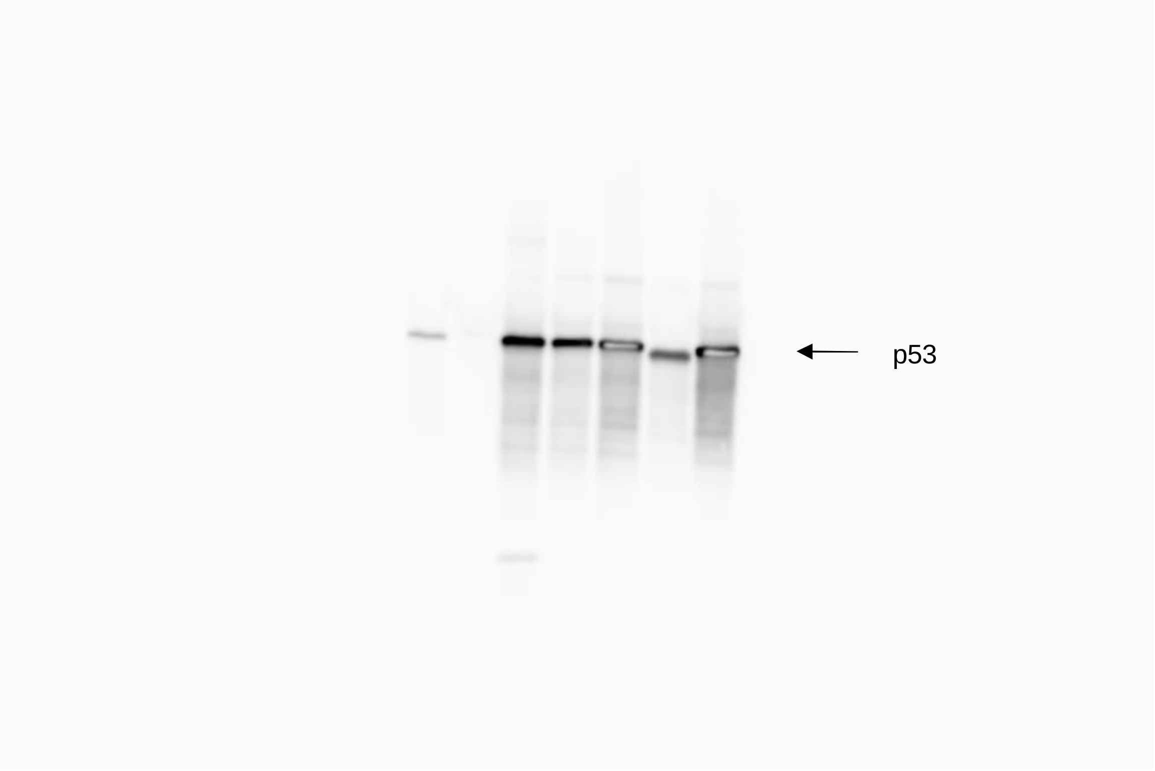
p53

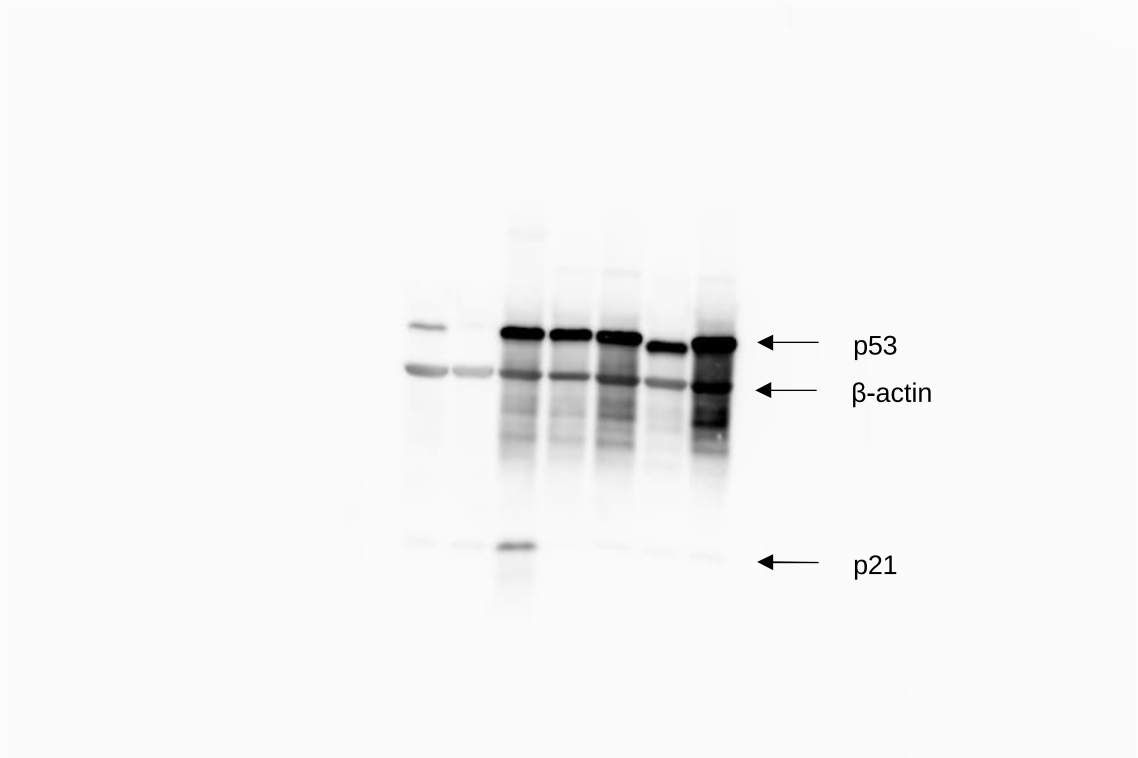

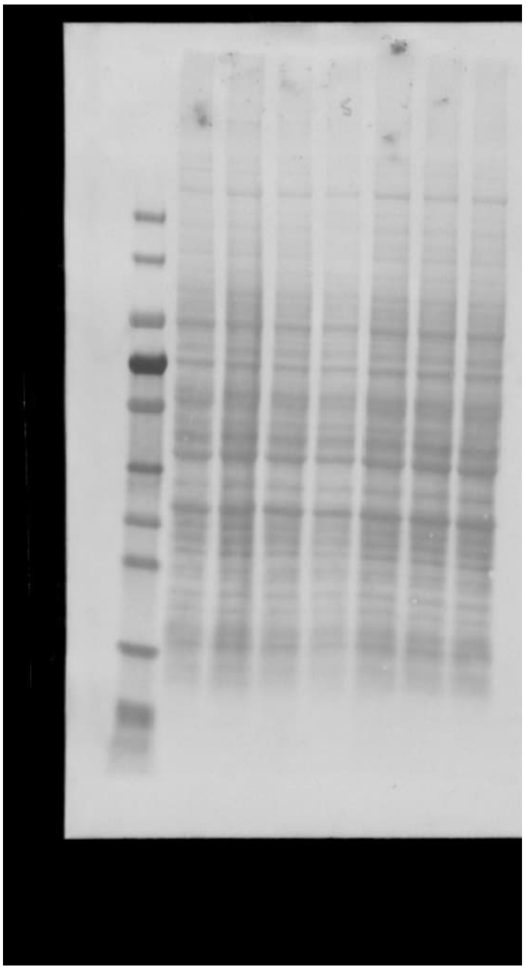

loading

Figure 3G:

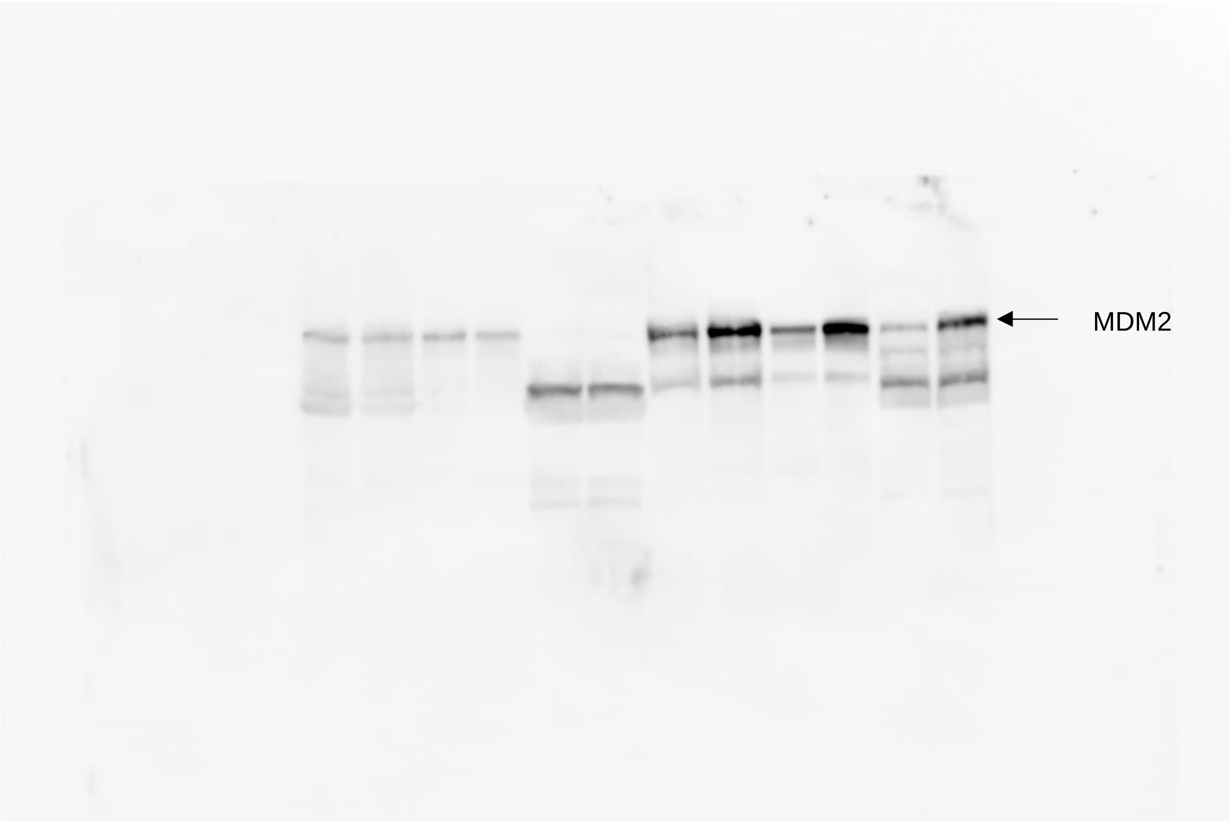

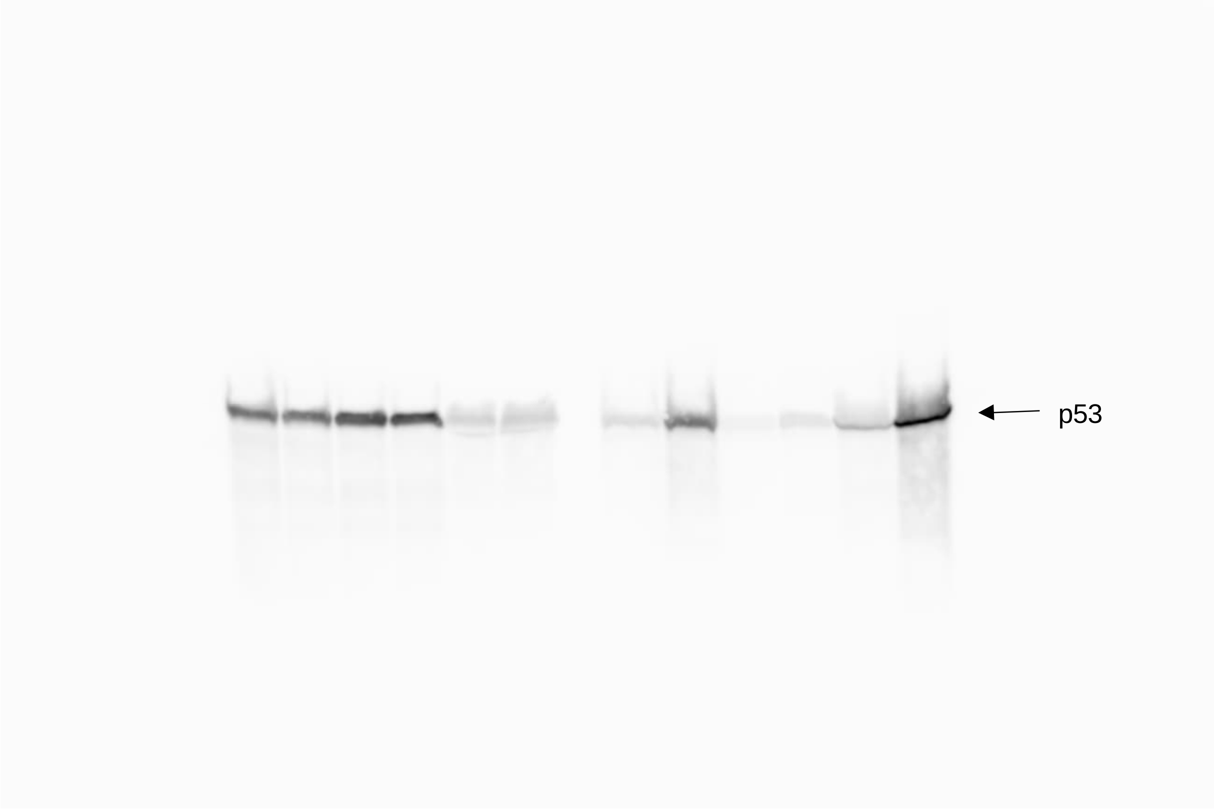

Figure 3G:

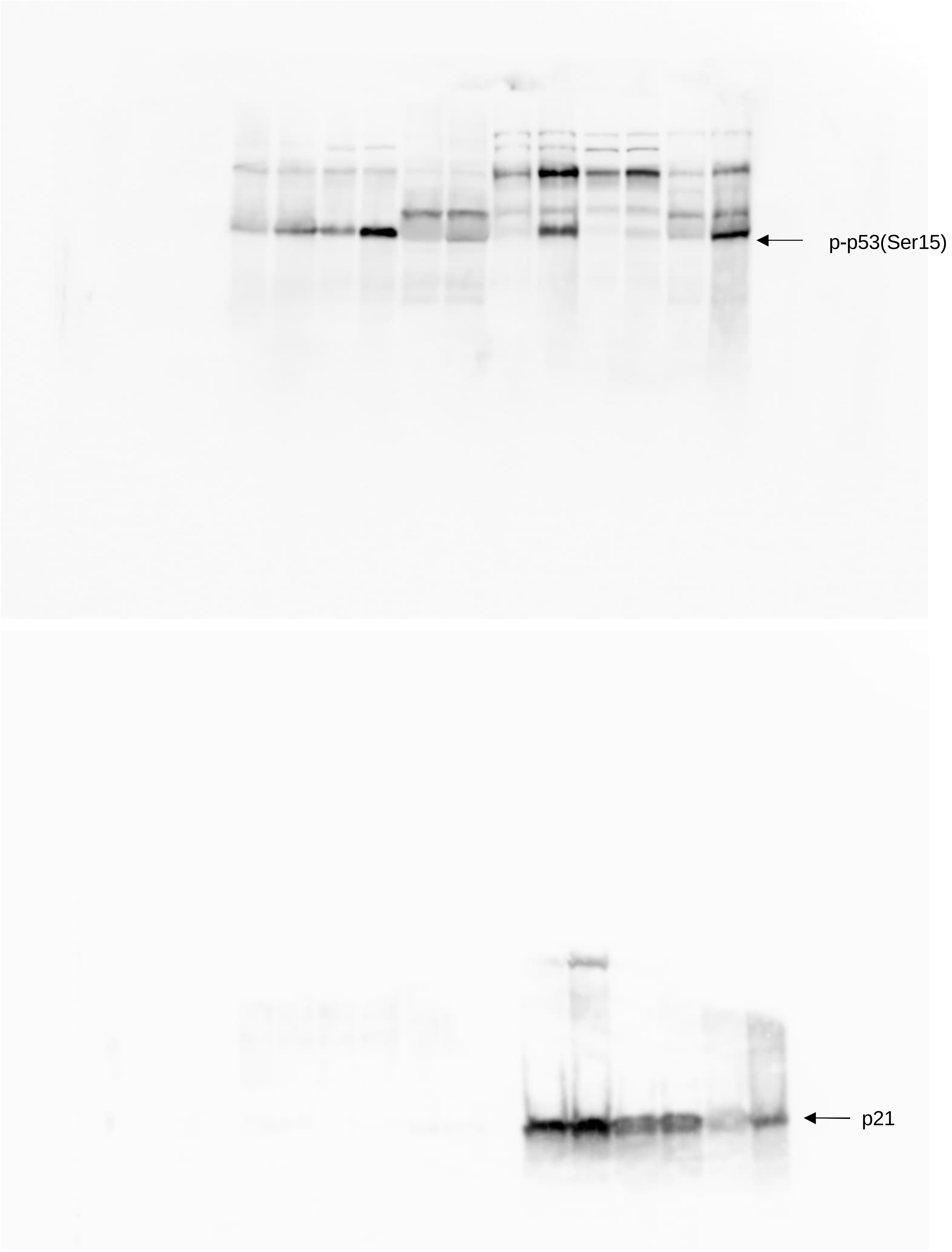

Figure 3G:

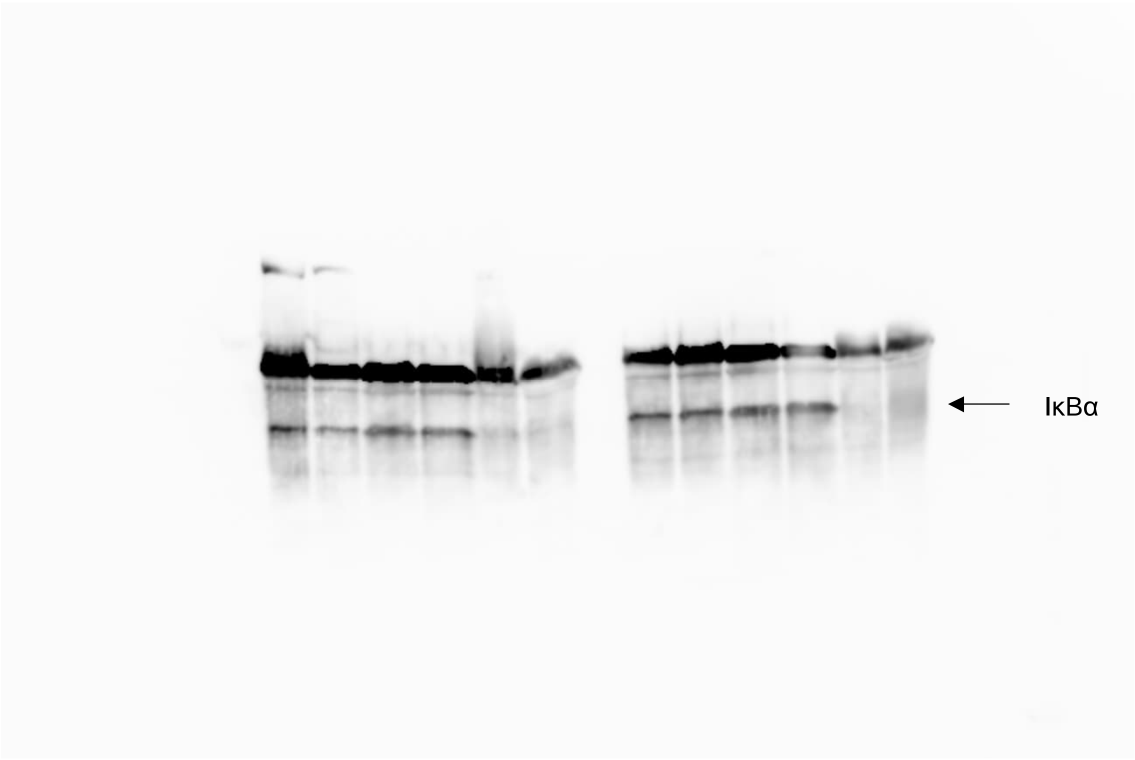

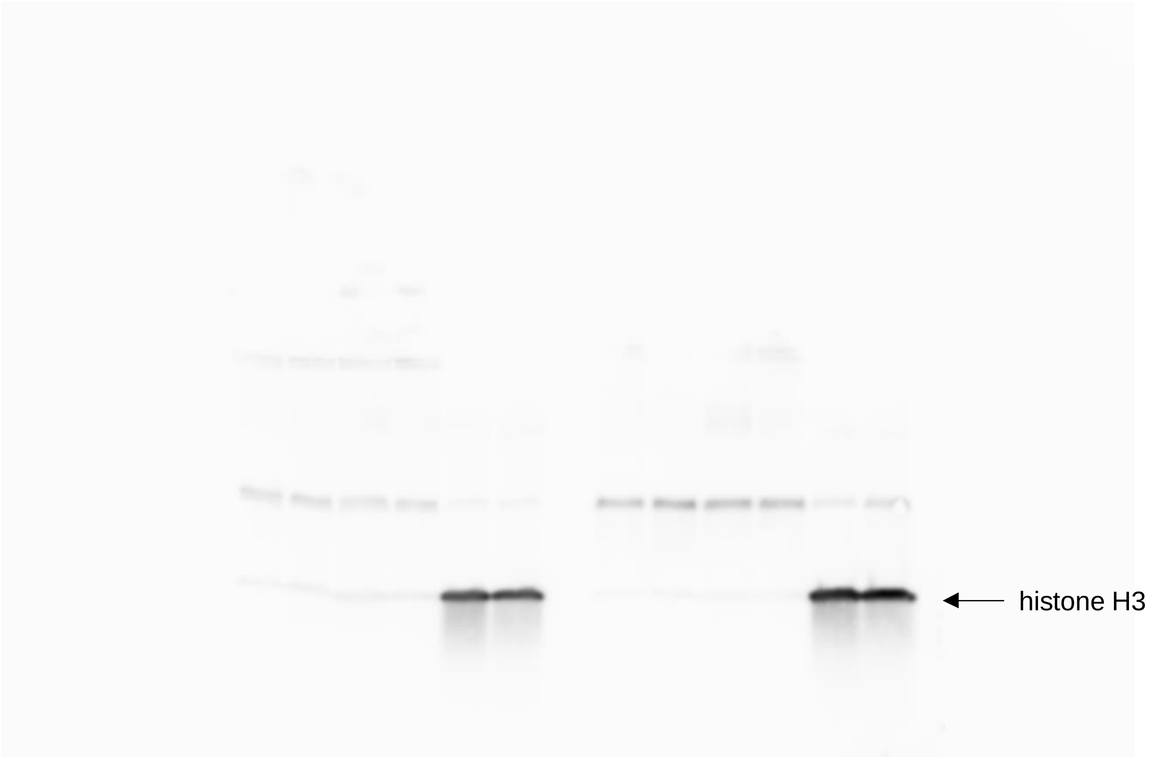

Figure 5B:

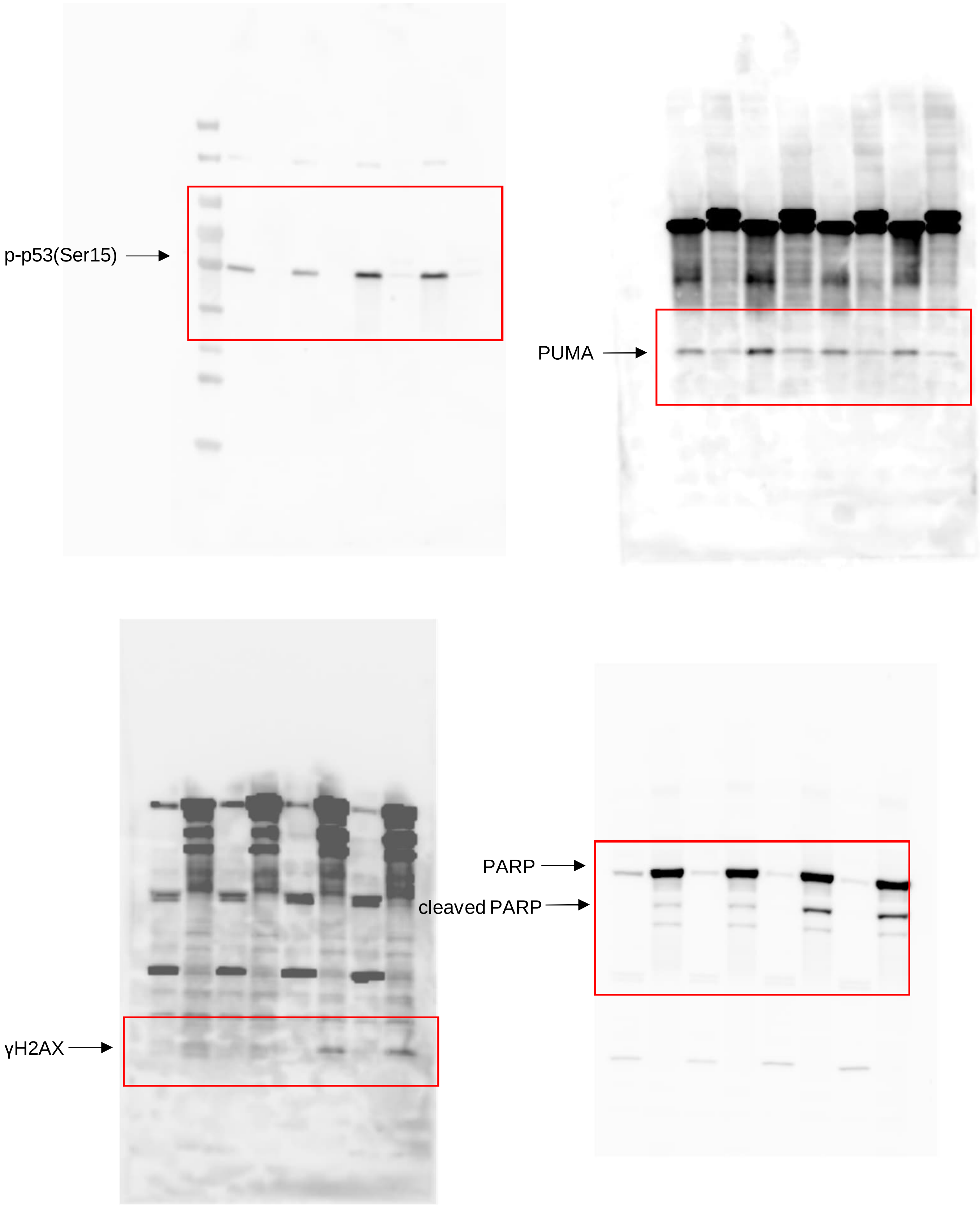

Figure 5B:

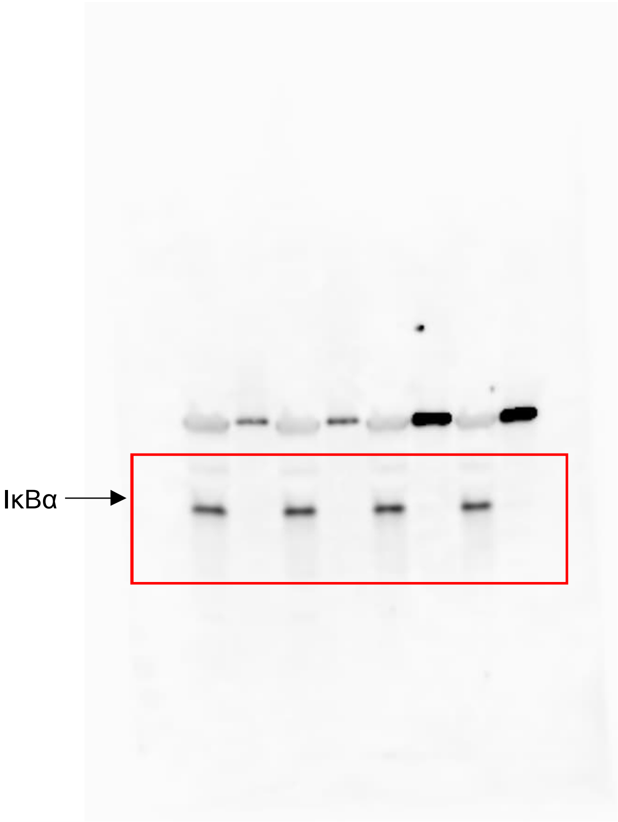

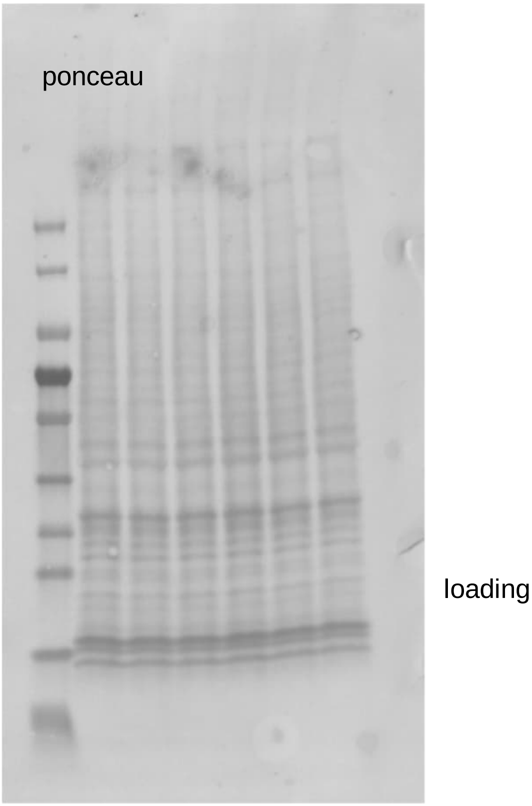

Figure 5E:

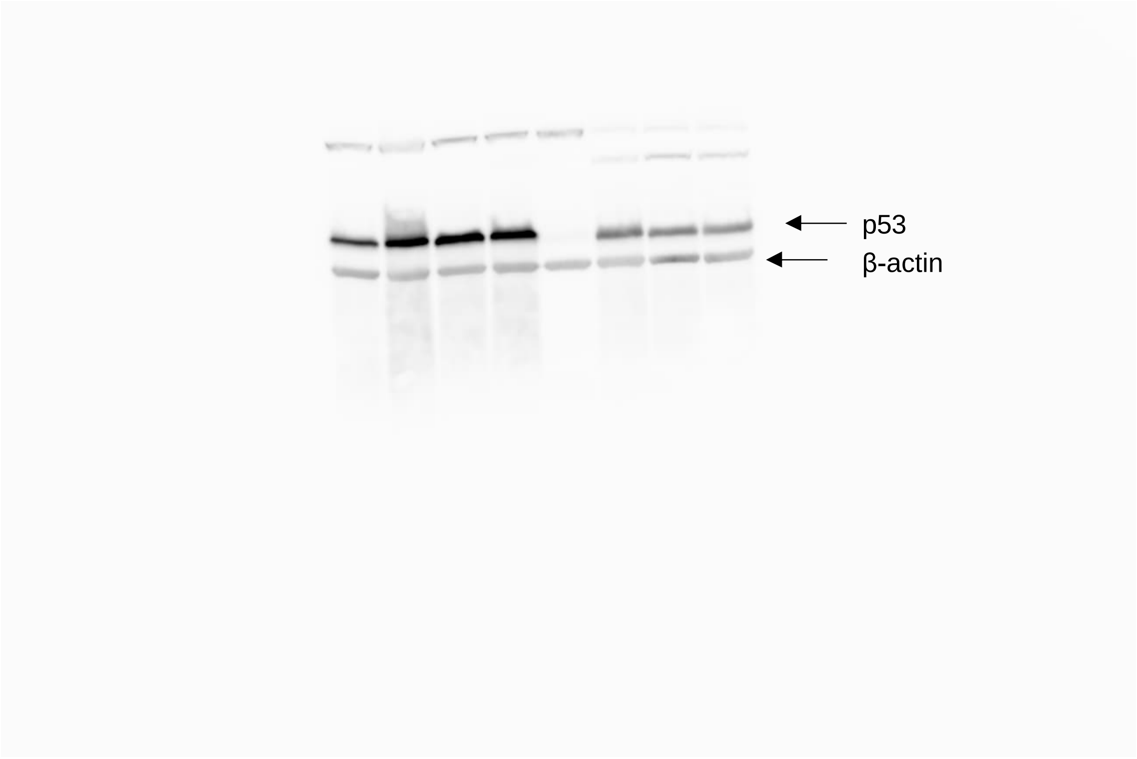

Figure 5E:

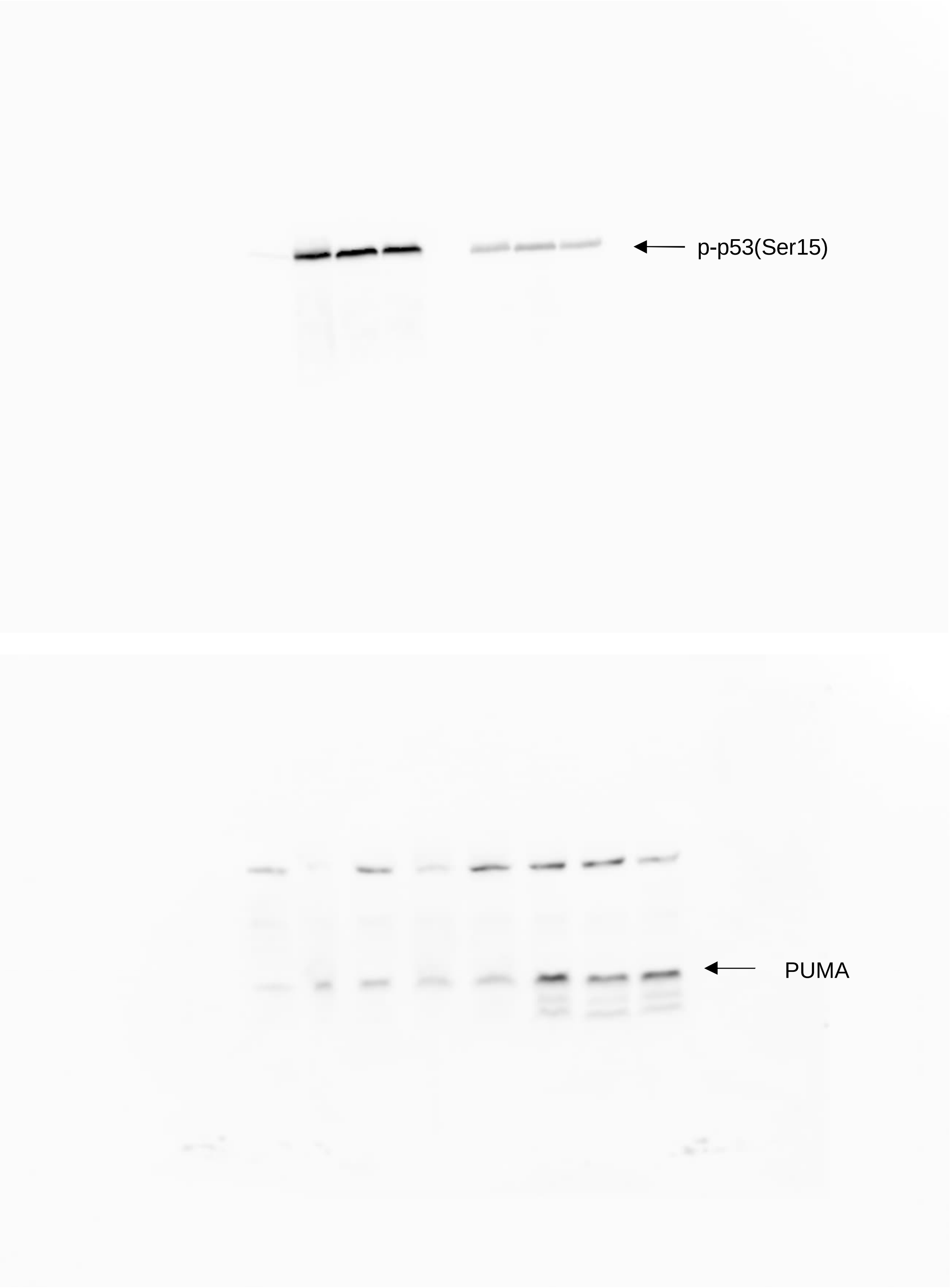

Figure 5E:

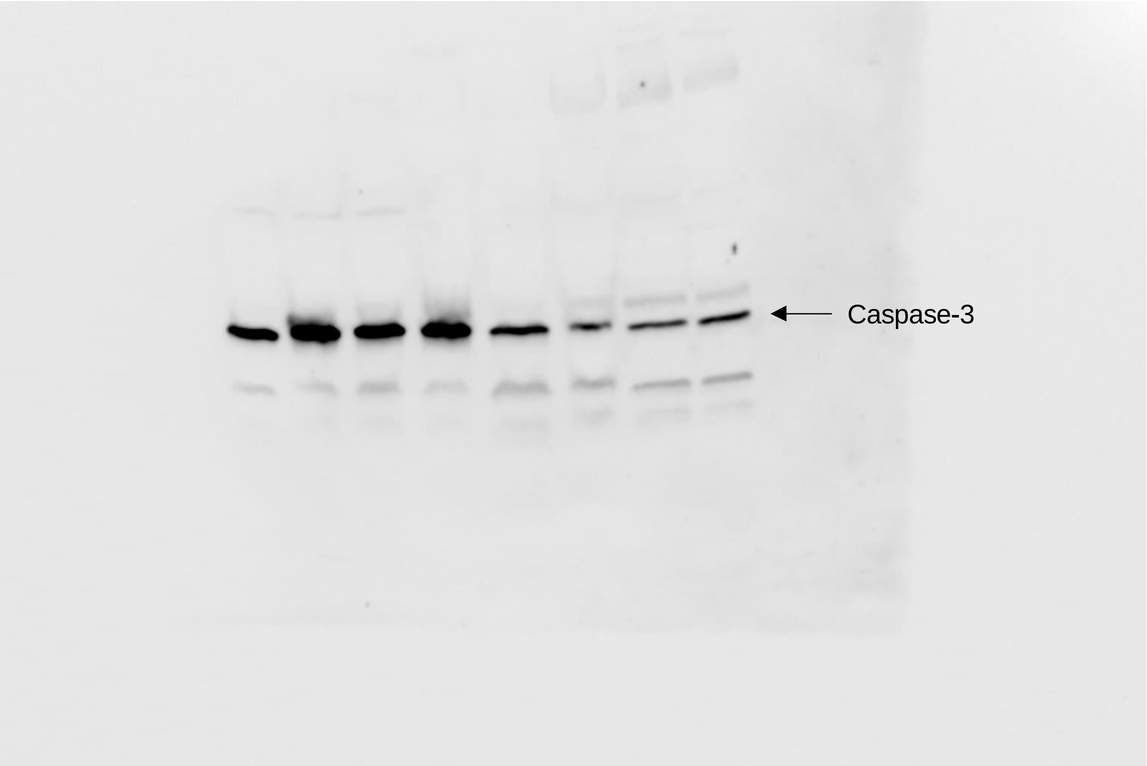

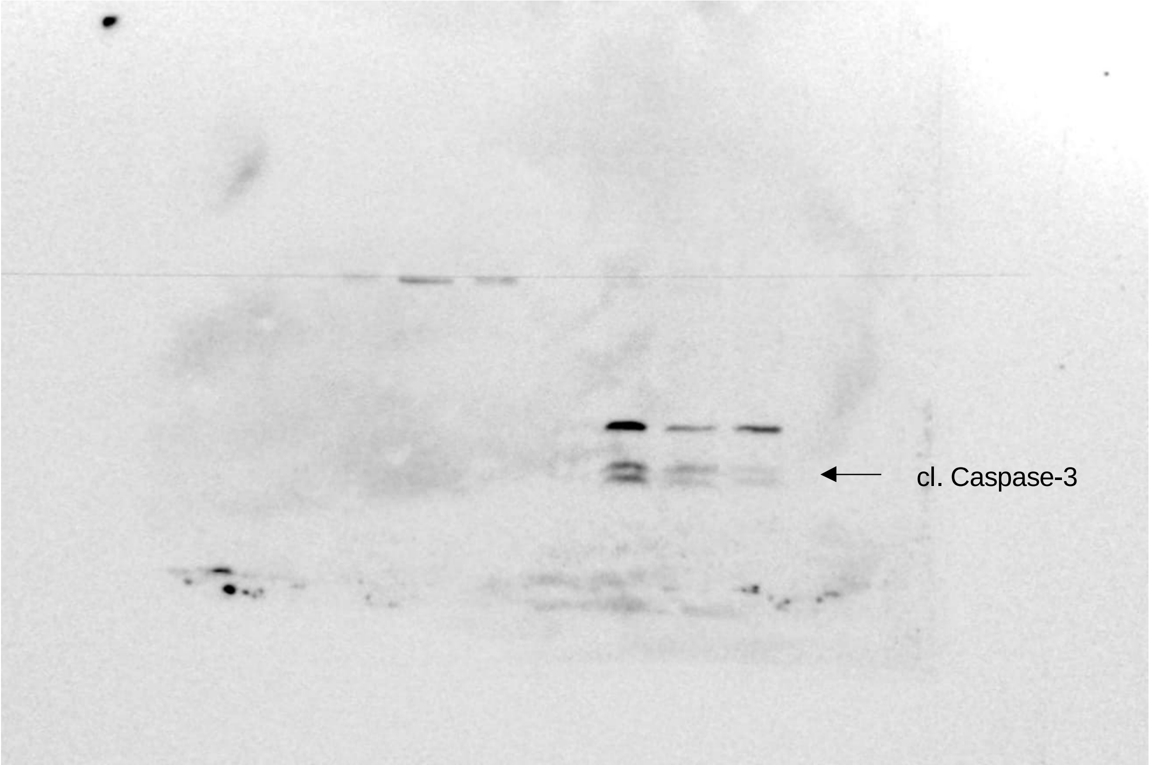

Figure 5E:

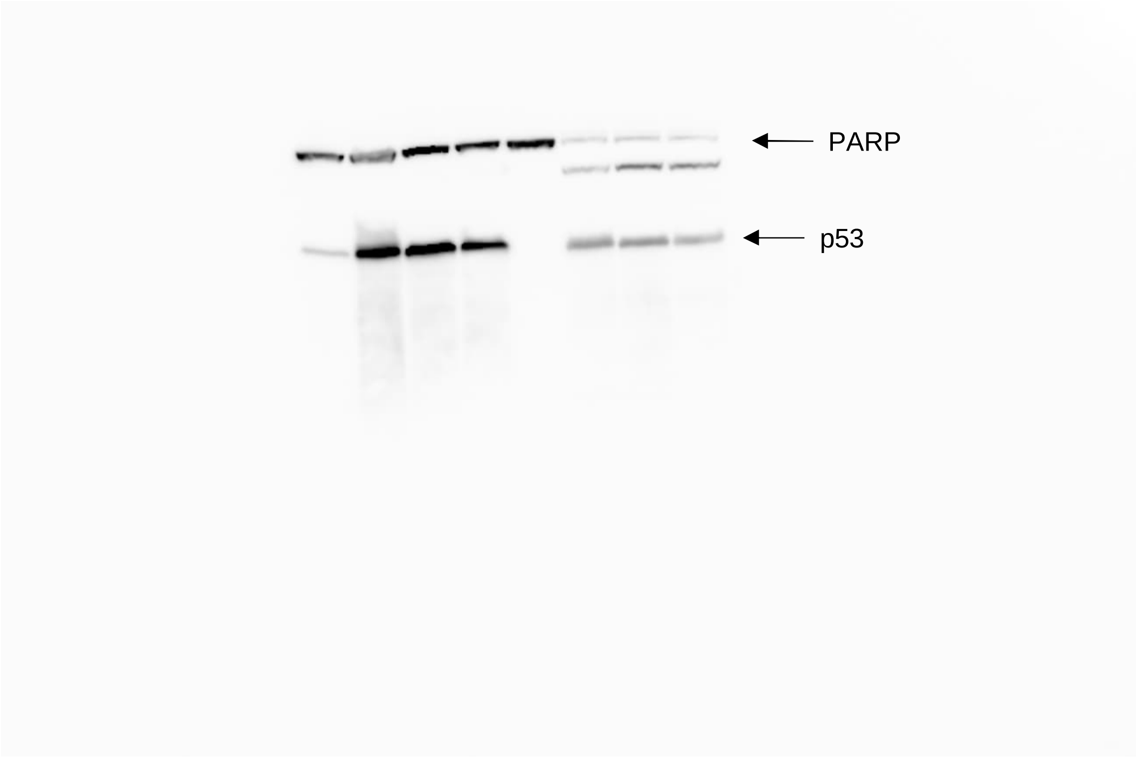

Figure S2:

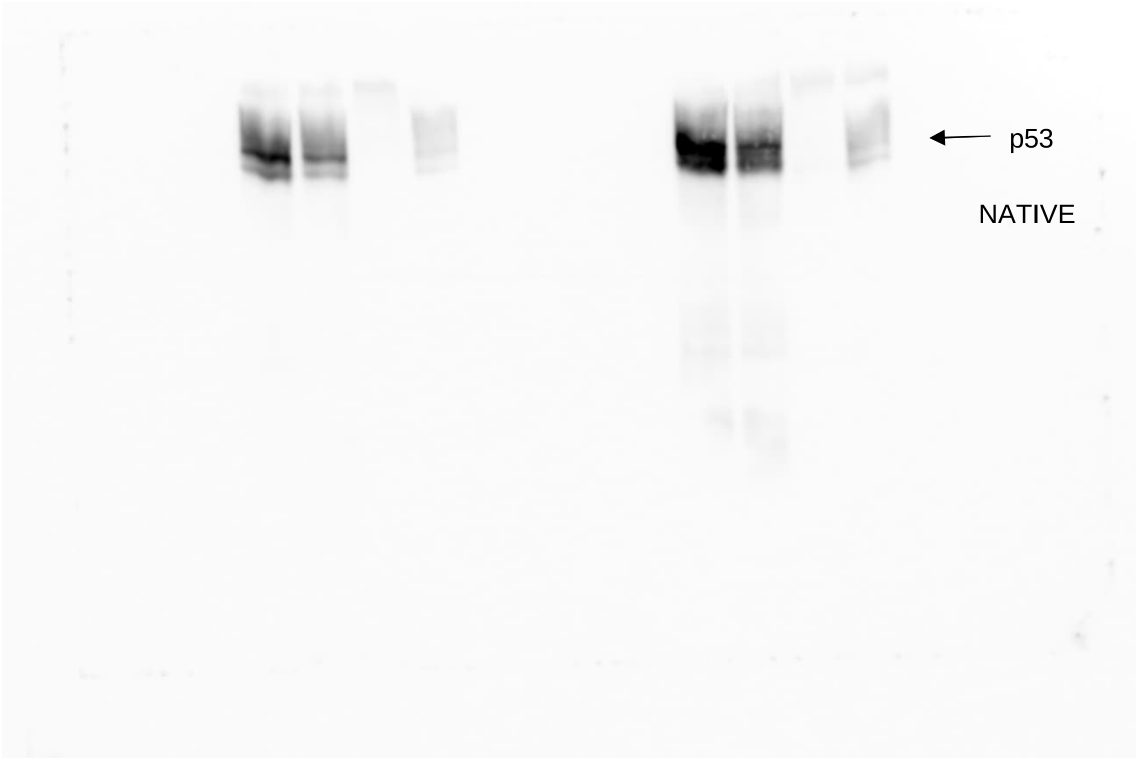

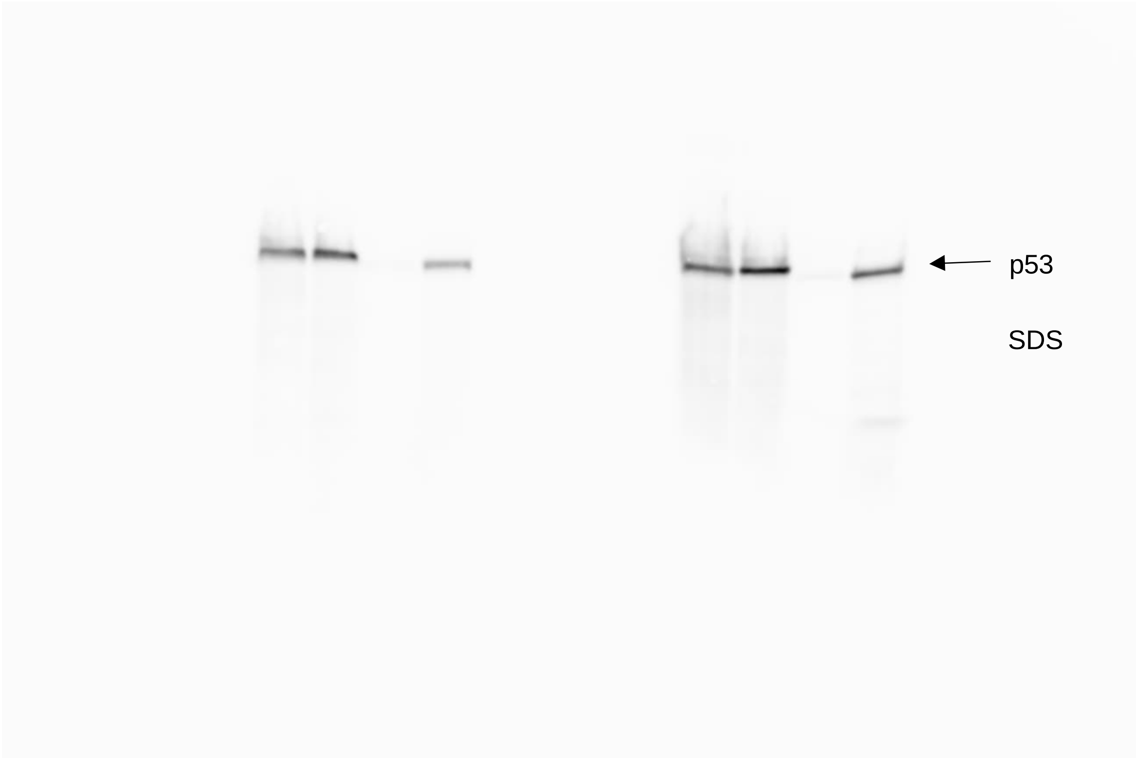

Figure S2:

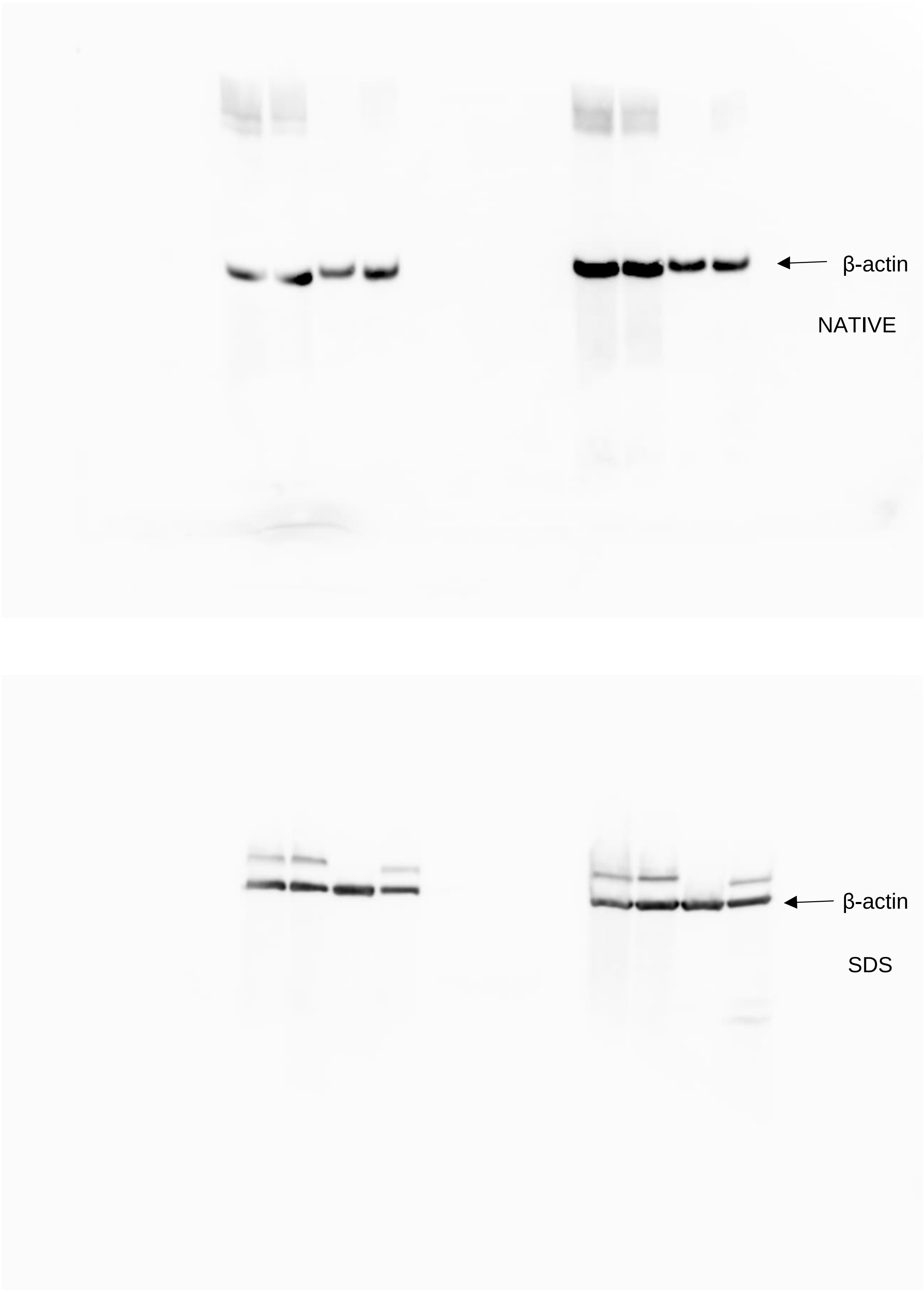

Figure S2:

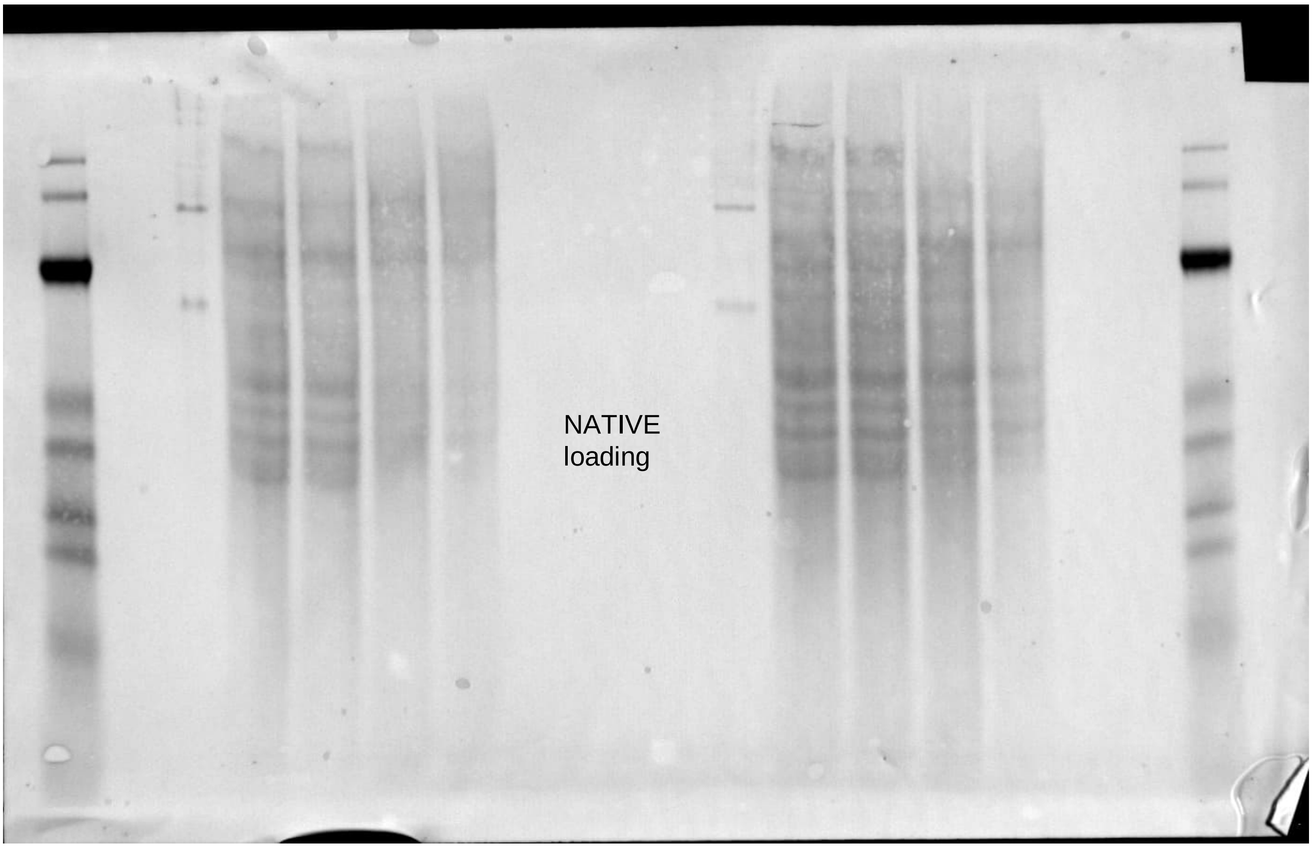

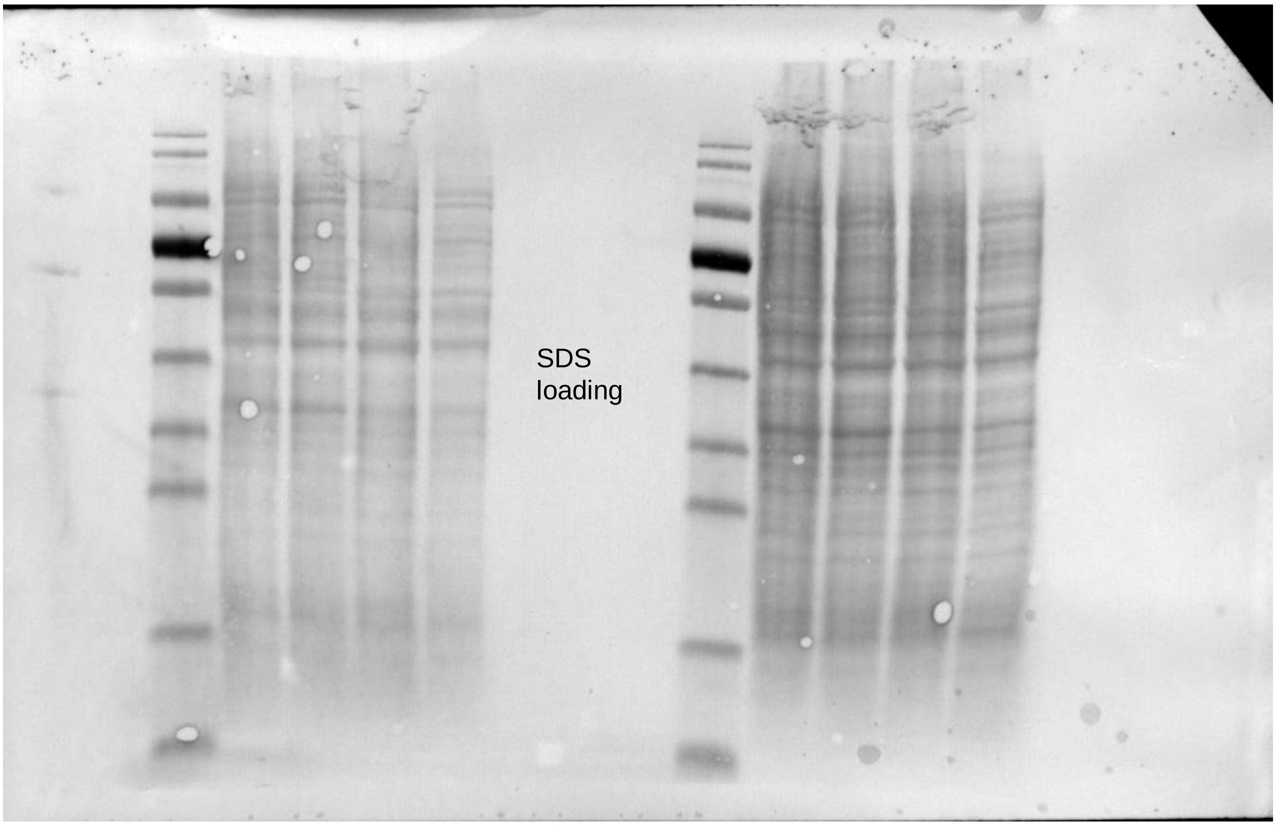

Figure S3:

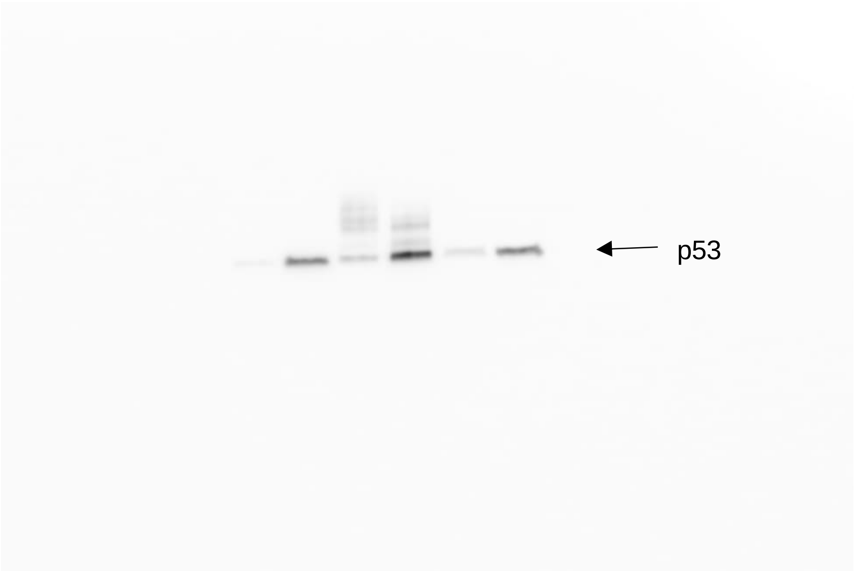

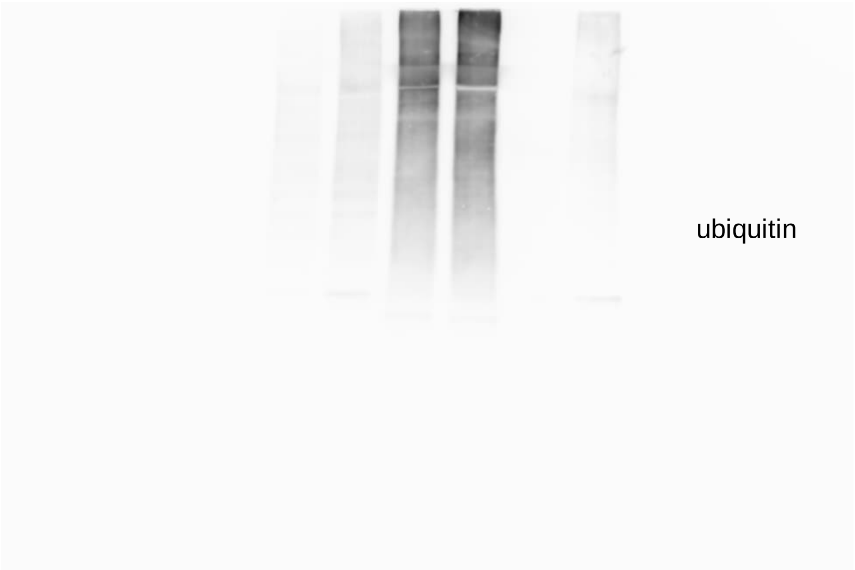

Figure S4:

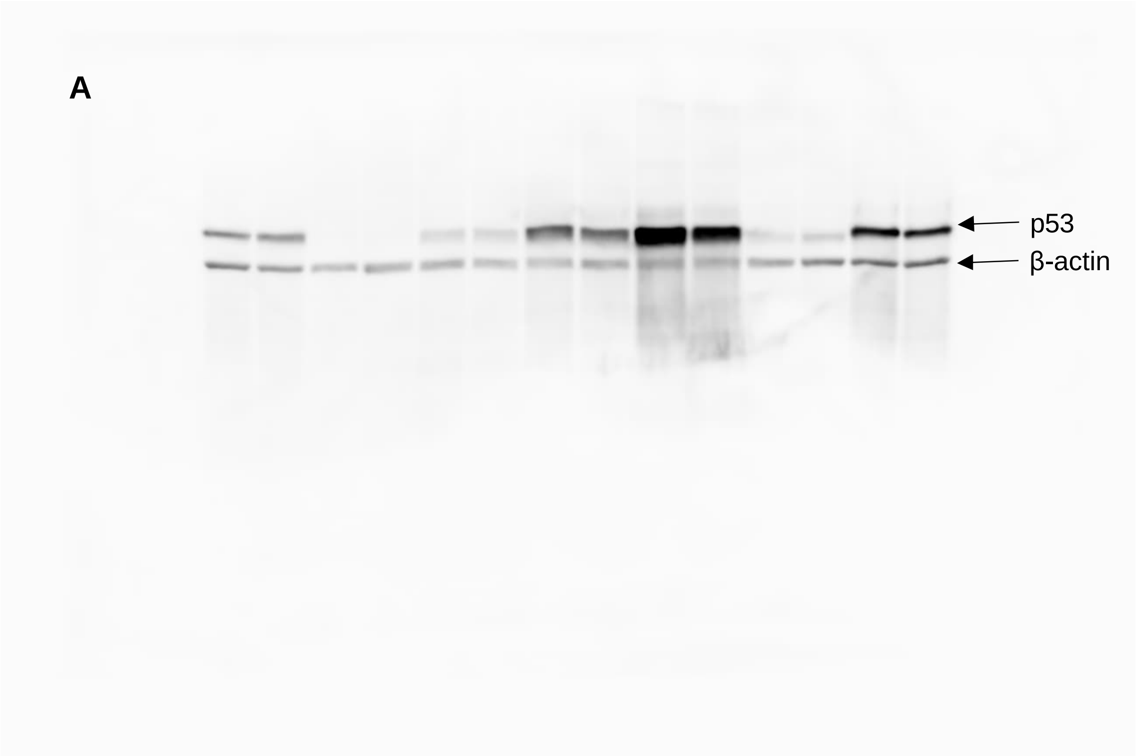

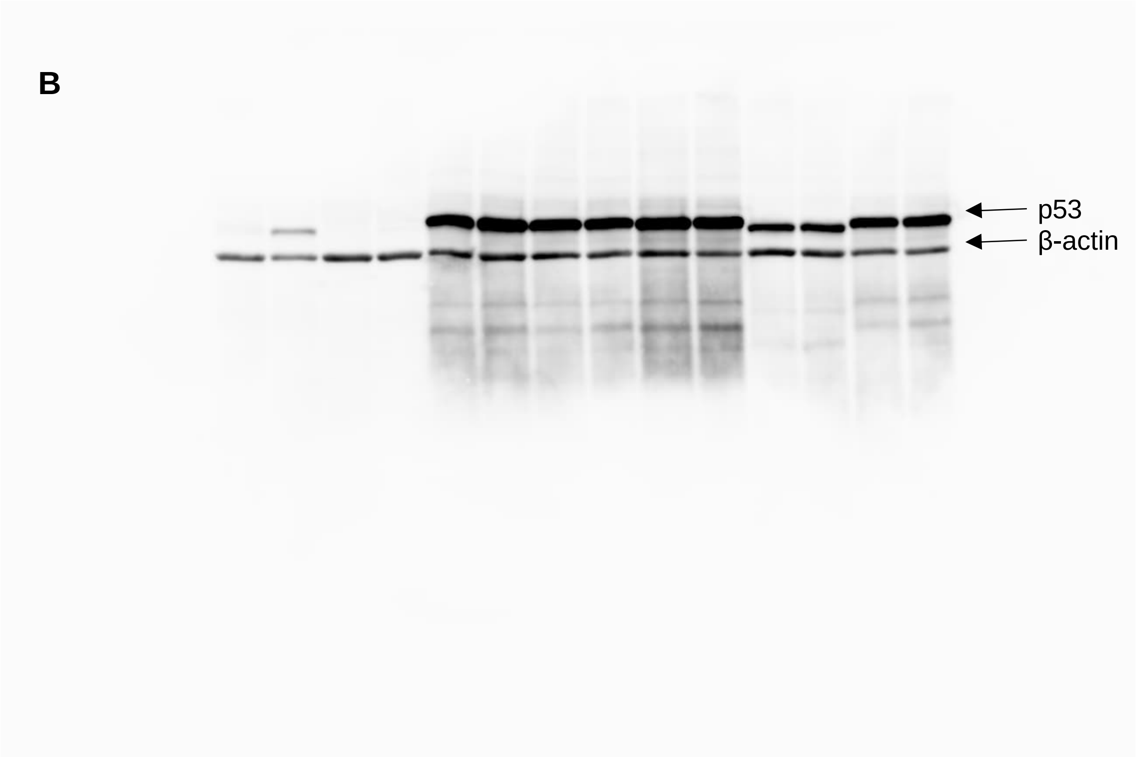

Figure S5B:

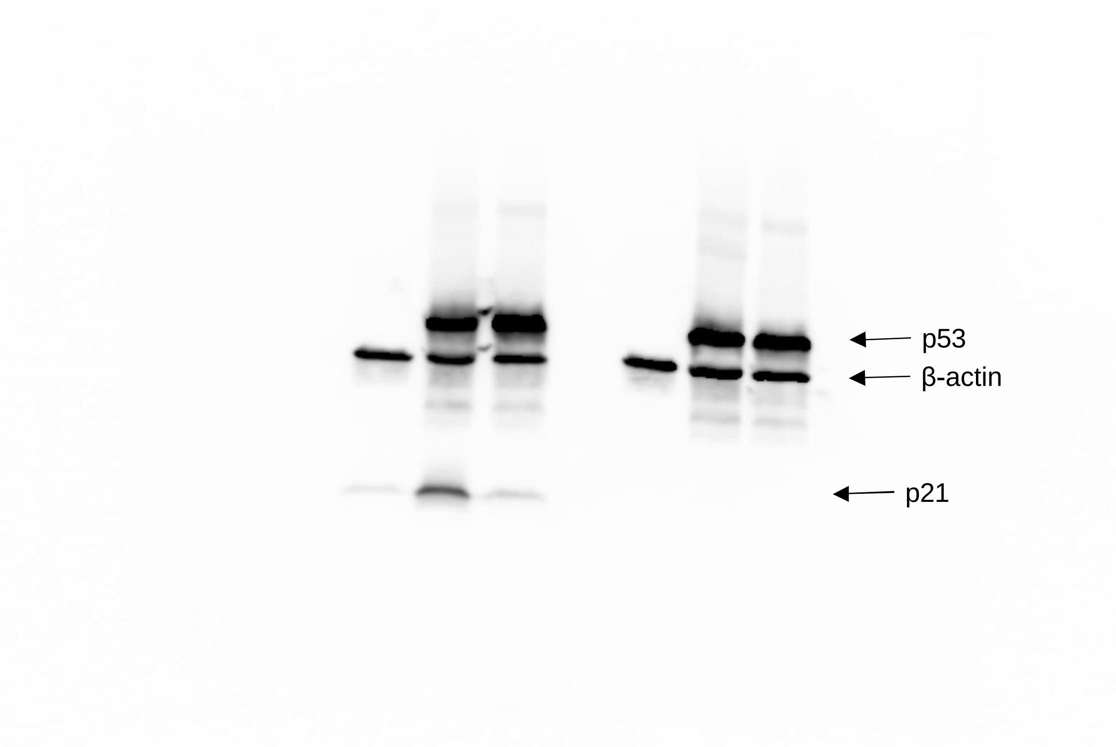

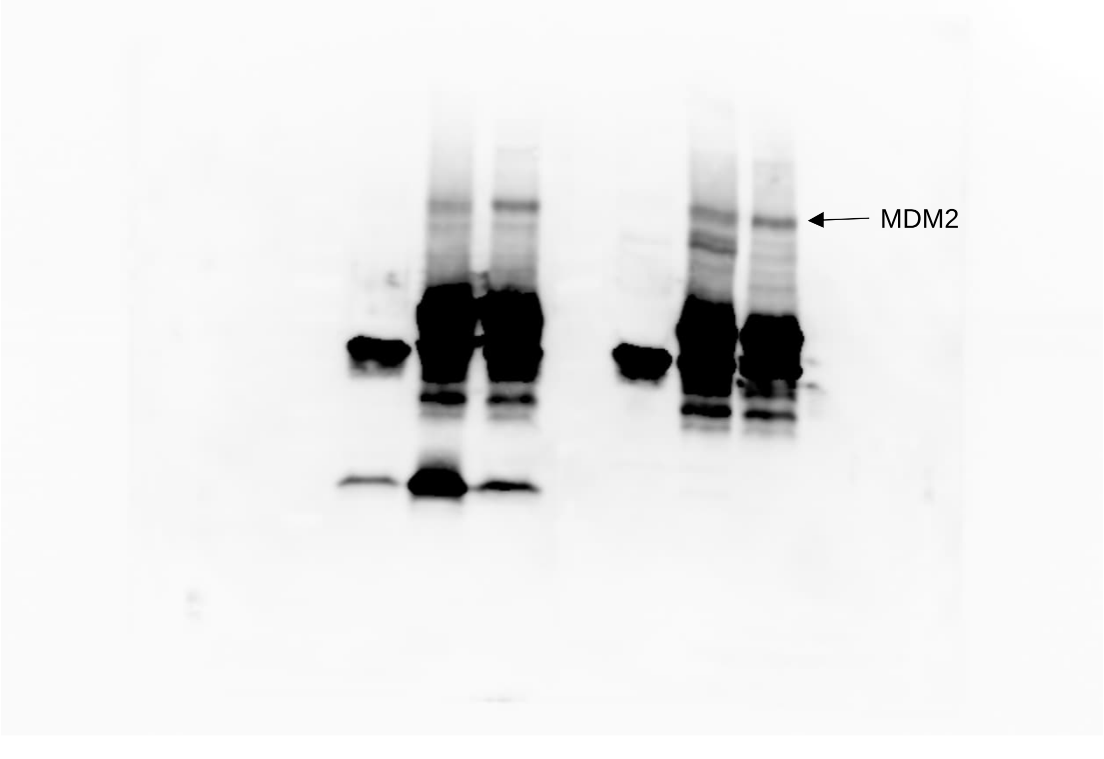

Figure S5D:

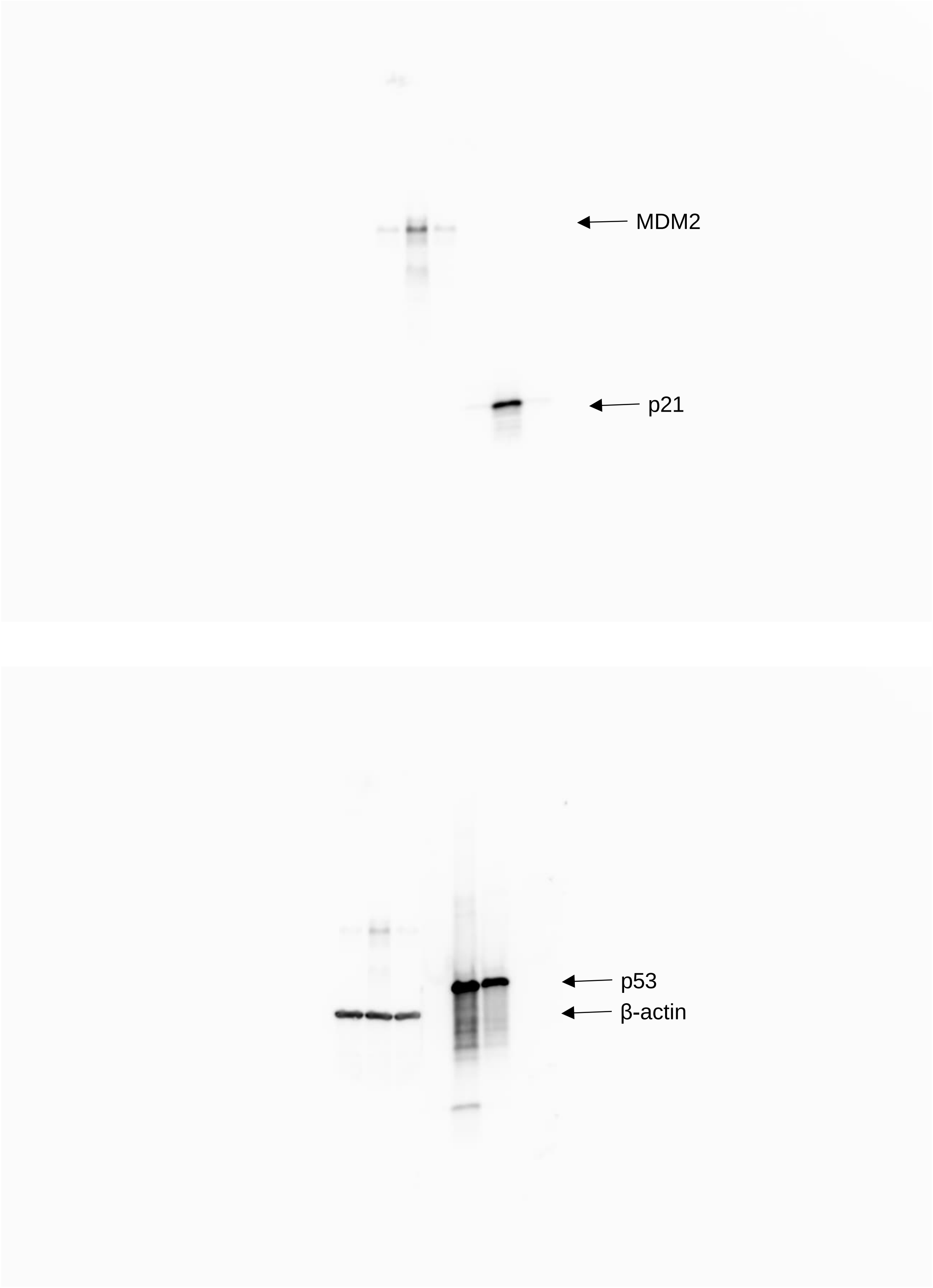

Figure S6:

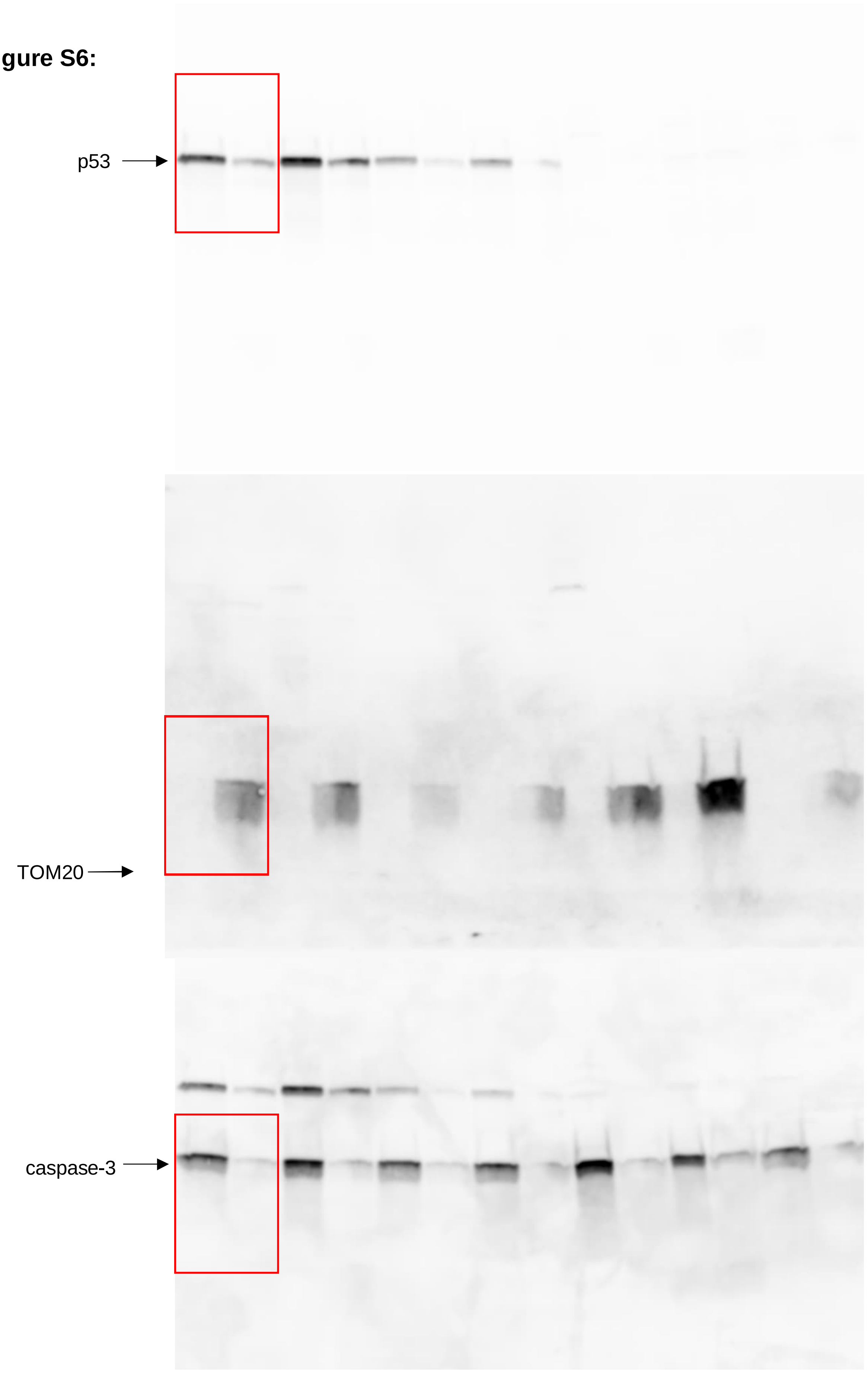

Supplement: Supplementary file 2 — Supplementary figures [file 41419_2023_6360_MOESM2_ESM.pdf]
